# Single-cell transcriptomics by clinical course of *Mycobacterium avium* complex pulmonary disease

**DOI:** 10.1038/s41598-024-66523-x

**Published:** 2024-07-08

**Authors:** Su-Young Kim, Sungmin Zo, Dae Hun Kim, Sung Jae Shin, Byung Woo Jhun

**Affiliations:** 1 Division of Pulmonary and Critical Care Medicine, Department of Medicine, Samsung Medical Center, Sungkyunkwan University School of Medicine, Seoul, South Korea; 2 Division of Respiratory and Critical Care Medicine, Department of Internal Medicine, Korea University Anam Hospital, Korea University College of Medicine, Seoul, South Korea; 3 Department of Microbiology, Institute for Immunology and Immunological Disease, Graduate School of Medical Science, Brain Korea 21 Project, Yonsei University College of Medicine, Seoul, South Korea

**Keywords:** *Mycobacterium avium* complex pulmonary disease, Disease progression, Treatment outcome, Immune profile, Single-cell RNA sequencing, Bacterial infection, Bacterial infection

## Abstract

*Mycobacterium avium* complex pulmonary disease (MAC-PD) has a heterogeneous clinical course. However, immune profiles associated with MAC-PD clinical course are limited. We performed single-cell RNA sequencing of peripheral blood mononuclear cells from 21 MAC-PD patients divided into three clinical courses: group A, spontaneous culture conversion; group B, stable disease without antibiotic treatment; and group C, progressive disease with antibiotic treatment. A lower proportion of NK cells and higher proportion of monocytes were noted in group C compared to combined groups A and B. The proportion of classical monocytes was higher in group C compared to groups A and B, while the proportion of non-classical monocytes decreased. EGR1, HSPA1A, HSPA1B, and CD83 were up-regulated in spontaneous culture conversion group A compared to progressive disease group C. Up-regulation of MYOM2 and LILRA4 and down-regulation of MT-ATP8, CD83, and CCL3L1 was found in progressive disease group C. PCBP1, FOS, RGCC, S100B, G0S2, AREG, and LYN were highly expressed in favorable treatment response compared to unfavorable response. Our findings may offer a comprehensive understanding of the host immune profiles that influence a particular MAC-PD clinical course and could suggest an immunological mechanism associated with the disease progression of MAC-PD.

## Introduction

Nontuberculous mycobacteria (NTM) are ubiquitous in natural environments^1^. NTM cause a variety of human diseases, most commonly chronic pulmonary disease (PD), and the global burden of NTM-PD is increasing^2^. Among NTM species, the *Mycobacterium avium* complex (MAC), mainly composed of *M. avium* and *M. intracellulare*, is the most common pathogen^3^. Two major species in MAC have a different epidemiology and pathogenesis. Regarding MAC-PD in East Asia, *M. avium* is the most common causative species in Japan, whereas *M. intracellulare* is the predominant organism in mainland China^4,5^. Additionally, household tap water was the main source of *M. avium* pulmonary infection, whereas *M. intracellulare* pulmonary infections were acquired from soil^6^. However, *M. avium* and *M. intracellulare* are infrequently differentiated in clinical practice. The guidelines from the Clinical and Laboratory Standards Institute for antimicrobial susceptibility test recommended the breakpoint for resistance to macrolide is identical within MAC species^7^. Also, the American Thoracic Society guidelines for treatment of NTM-PD recommended treatment regimens for MAC, *M. kansasii*, *M. xenopi*, *M. abscessus-*PD^8,9^. Macrolide-based combination antibiotic therapy is recommended as the initial therapy for MAC-PD, and the guidelines recommend different antibiotic regimens according to clinical phenotype, not causative etiology: intermittent, three-times-weekly oral administration of three drugs [macrolide, ethambutol, and rifamycin] for non-cavitary nodular bronchiectatic MAC-PD versus daily oral drugs with or without administration of parenteral drugs, such as streptomycin or amikacin, for cavitary MAC-PD^8,9^.

The most notable aspect of MAC-PD is its heterogeneous clinical course. Approximately 30% to 40% of MAC-PD patients show spontaneous improvement or remain stable for several years without antibiotic therapy^10^, whereas other MAC-PD patients show deterioration leading to destruction of the lung parenchyma^11^. Additionally, treatment outcomes of MAC-PD are diverse. Despite the long treatment duration, MAC-PD is refractory in more than 30% of patients^12^. Unfortunately, a few clinical indicator of disease deterioration has been identified. Data on the heterogeneity of the disease course or treatment responses are also limited^10^, possibly because MAC-PD is a multifactorial disease influenced by host immunity, microbiological factors, and environmental exposure.

As MAC-PD occurs in only a subset of patients with structural lung disease and many patients have characteristic phenotypes such as ‘*Lady Windermere syndrome*’ rather than disseminated infection^13,14^, it is important to assess host immunity. Immunologic and genetic studies have implicated several cytokines and genes, including interferon (IFN)-γ and Toll-like receptor (TLR), in MAC-PD pathogenesis, suggesting that NTM infection exerts an immunomodulatory effect on the host^15–17^. However, the studies have limitations, with inconsistent results. There is also no evidence on whether these genes and cytokines are truly predisposing factors or simply a result of MAC-PD.

Transcriptome profiling using single-cell RNA sequencing (scRNA-seq) enables the identification of transcriptional similarities and differences within known and novel cell populations^18^. scRNA-seq also facilitates assessment of the heterogeneity of gene expression according to disease status^19,20^.

The clinical course of MAC-PD varies from patient to patient, but can be broadly divided into cases of spontaneous negative conversion, stable course, and cases treated when the disease progresses. Immune profiles will also vary depending on the clinical course. If there is a predictive factor that can predict disease progression or treatment response before starting treatment, it can be used as a reference for long-term antibiotic treatment. Therefore, we performed scRNA-seq of peripheral blood mononuclear cells (PBMCs) from MAC-PD patients to identify immune profiles associated with disease progression or treatment outcome of MAC-PD.

## Results

### Patient population

The median age of the 21 patients was 60.0 years, and 17 (81%) patients were female (Table 1). Three (14%) patients had a previous history of pulmonary tuberculosis while the majority (95%) had underlying bronchiectasis. Twelve patients (57%) had *M. avium*, and eight (38%) patients had *M. intracellulare*. All patients except for one presented with the nodular bronchiectatic radiologic form of MAC-PD.

**Table 1:** Clinical characteristics of study patients (n = 21).

Characteristics	Value
Age, years	60.0 (51.5–68.0)
Sex, FEMALE	17 (81)
Body mass index, kg/m^2^	21.0 (20.4–22.6)
Never smoker	17 (81)
Underlying disease	
Previous pulmonary tuberculosis	3 (14)
Obstructive pulmonary disease or asthma	0 (0)
Chronic pulmonary aspergillosis	1 (5)
Bronchiectasis	20 (95)
Sputum acid-fast bacilli smear positivity	5 (24)
Etiology	
* M. avium*	12 (57)
* M. intracellulare*	8 (38)
* M. avium* and *M. intracellulare* mixed infection	1 (5)
Radiological form of MAC-PD	
Nodular bronchiectatic form	20 (95)
Fibrocavitary form	1 (5)
Patient group according to disease progression	
Group A, Spontaneous conversion	5 (24)
Group B, Stable disease	7 (33)
Group C, Progressive disease	9 (43)
Subgroup C-1, favorable response	3 (33)
Subgroup C-2, unfavorable response	3 (33)
Subgroup C-x, unclassified	3 (33)

Data are presented as n (%) or the median (interquartile range).

**Figure 1:**
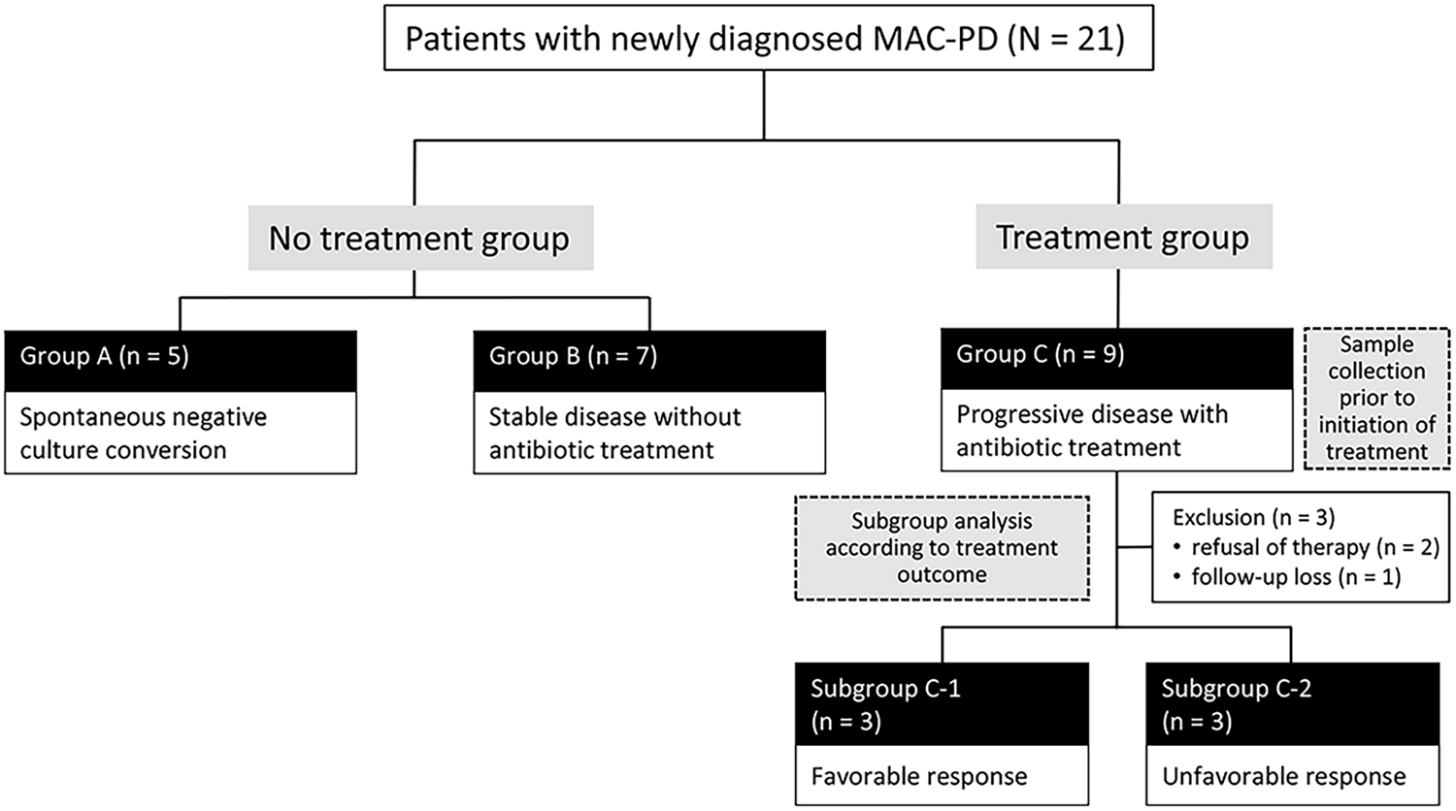
Study population.

Of the 21 patients, five patients achieved spontaneous negative culture conversion (group A, spontaneous conversion). In seven patients, the disease did not show progression for several years after diagnosis (group B, stable disease). The remaining nine patients had progressive disease that required initiation of antibiotic treatment (group C, progressive disease). In group C, two patients refused continuing antibiotic therapy; of the other seven group C patients who received antibiotic therapy, three achieved negative culture conversion (favorable response, subgroup C-1), three failed culture conversion (unfavorable response, subgroup C-2), and one did not complete treatment due to follow-up loss (Fig. 1, Supplementary Table S1 online).

### Annotation of five main types of single cells

**Figure 2:**
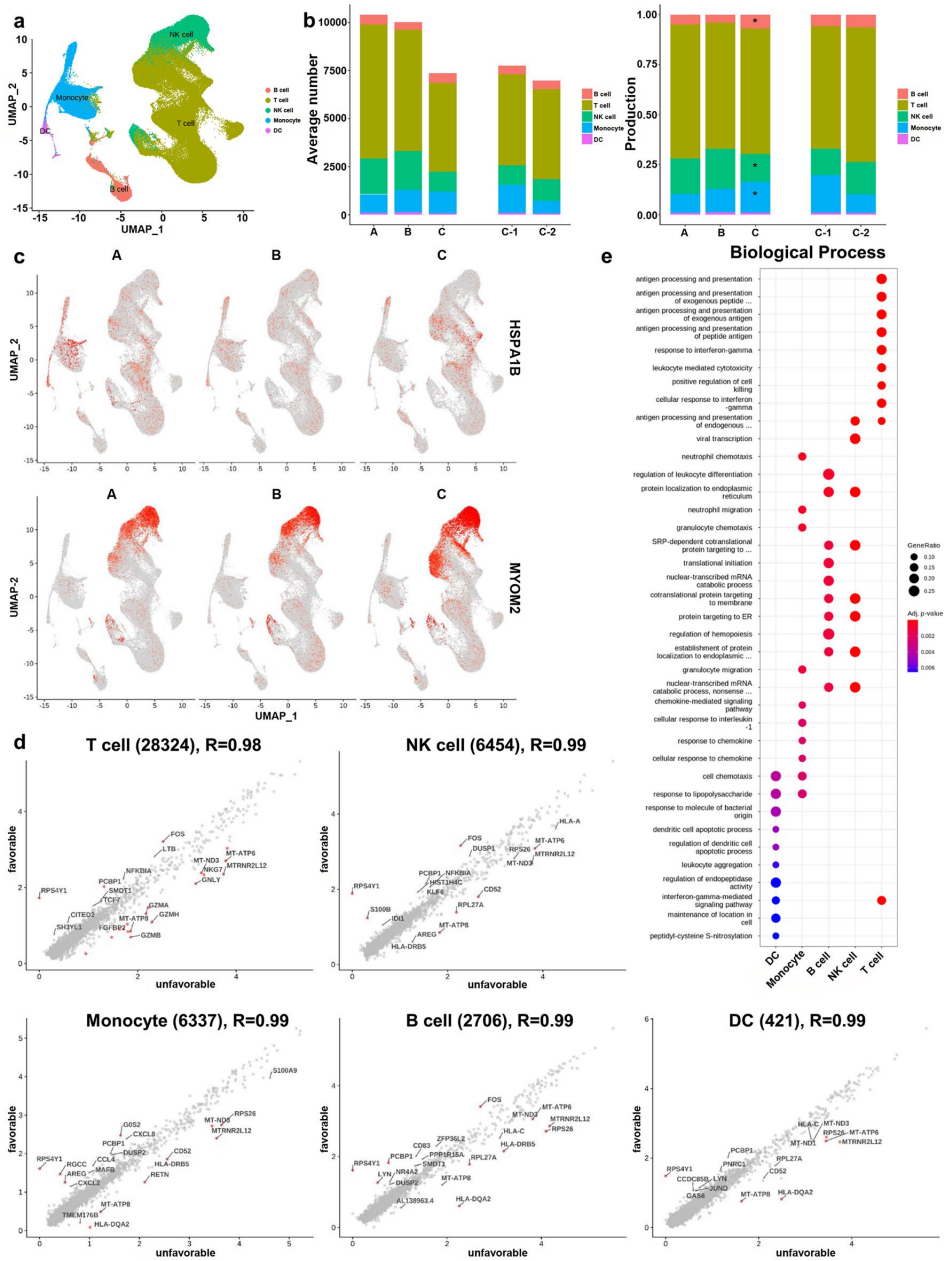
Single-cell transcriptomes of PBMCs from MAC-PD patients. (**a**) UMAP plot of single-cell profiles with each cell color-coded by cell type. (**b**) Average cell number and proportion of five main cells from PBMCs of each group. (**c**) UMAP plot of representative gene expression patterns for HSPA1B (group A) and MYOM2 (group C), split by group. (**d**) Scatter plot showing DEGs between subgroup C-1 and C-2. Each dot indicates an individual gene, colored by red when a gene had a significant difference in expression. (**e**) Functional enrichment analysis of DEGs in subgroup C-1 compared to subgroup C-2. Dot plot showing the most significant GO terms in each cell type. The top 10 GO terms were selected from each cluster. Dot size represents the gene ratio included in each GO term. Color gradient of the dots represents the adjusted p-values of each enriched GO term.

Totally, 189,779 cells were clustered into 31 cell populations via the uniform manifold approximation and projection (UMAP) method (Supplementary Fig. S1a online). Five cell types were annotated with distinct markers as follows (from most to least abundant): T cells, natural killer (NK) cells, monocytes, B cells, and dendritic cells (DCs) (Fig. 2a, Supplementary Fig. S1b–d online). A lower proportion of NK cells and a higher proportion of monocytes were observed in group C compared to combined groups A and B (Fig. 2b, Supplementary Fig. S1e,f online, and Supplementary Tables S3 and S4 online). In subgroup analysis of 12 M*. avium* patients, those differences in all MAC-PD patients were not shown (Supplementary Table S6). In subgroup analysis of eight *M. intracellulare* patients, the proportion of NK cells, monocytes, and non-classical monocytes was not different among three groups, but, the proportion of classical monocytes was higher in group C compared to combined groups A and B. Also, a lower proportion of naïve CD4 T cells and Tregs was noted in group C compared to combined groups A and B (Supplementary Table S7). In subgroup analysis of 16 MAC-PD patients excluding male and non-bronchiectasis/non-nodular bronchiectatic MAC-PD patients, those differences in all MAC-PD patients was shown consistently (Supplementary Table S8).

### Transcriptional signatures of five cell types associated with clinical features

To identify indicators of disease progression and treatment response, we first looked for differentially expressed genes (DEGs) in groups A and B. Only three genes were up- or down-regulated (|fold change|≥ 2) (Supplementary Fig. S2a online). Genes RPS4Y1 in most cells and CCL4L2 in NK cells were up-regulated in group B, while HSPA1B in monocytes was down-regulated (Fig. 2c). However, RPS4Y1 is reportedly a gender-specific transcript marker on the Y chromosome^21^ and thus was excluded from interpretation of results. Second, we compared groups A and C, finding six up- or down-regulated genes (Supplementary Fig. S2b online). MYOM2 in NK cells and LILRA4 in DCs were up-regulated in group C (Fig. 2c) whereas EGR1, HSPA1A, and HSPA1B in monocytes and CD83 in B cells were down-regulated in group C. Third, comparison of groups B and C identified only four up- or down-regulated genes, with MYOM2 in NK cells up-regulated in group C (Fig. 2c and Supplementary Fig. S2c online) and CD83 in B cells, MT-ATP8 in monocytes and NK cells, and CCL3L1 in monocytes down-regulated in group C. In the comparison between groups A and B, there were few DEGs, so the two groups were analyzed together. Fourth, we combined groups A and B as a no-treatment group and identified disease progression-associated changes in genes for each cell type when compared to the treatment group (group C) (Supplementary Fig. S2d online). MYOM2 in NK cells was specifically up-regulated in treatment group C, and CD83 in B cells and MT-ATP8 in monocytes were down-regulated in group C (Supplementary Fig. S2e online). Lastly, to identify indicators of treatment response, we compared the subgroups C-1 and C-2 (Fig. 2d). PCBP1, FOS, RGCC, S100B, G0S2, AREG, and LYN were up-regulated in group C-1. Functional enrichment analysis of upregulated DEGs in subgroup C-1 showed significant up-regulation of antigen processing and presentation, leukocyte-mediated cytotoxicity, and cellular response to IFN-γ in T cells, and protein localization to endoplasmic reticulum (ER) in NK cells and B cells (Fig. 2e).

### Analysis of T cell subsets

**Figure 3:**
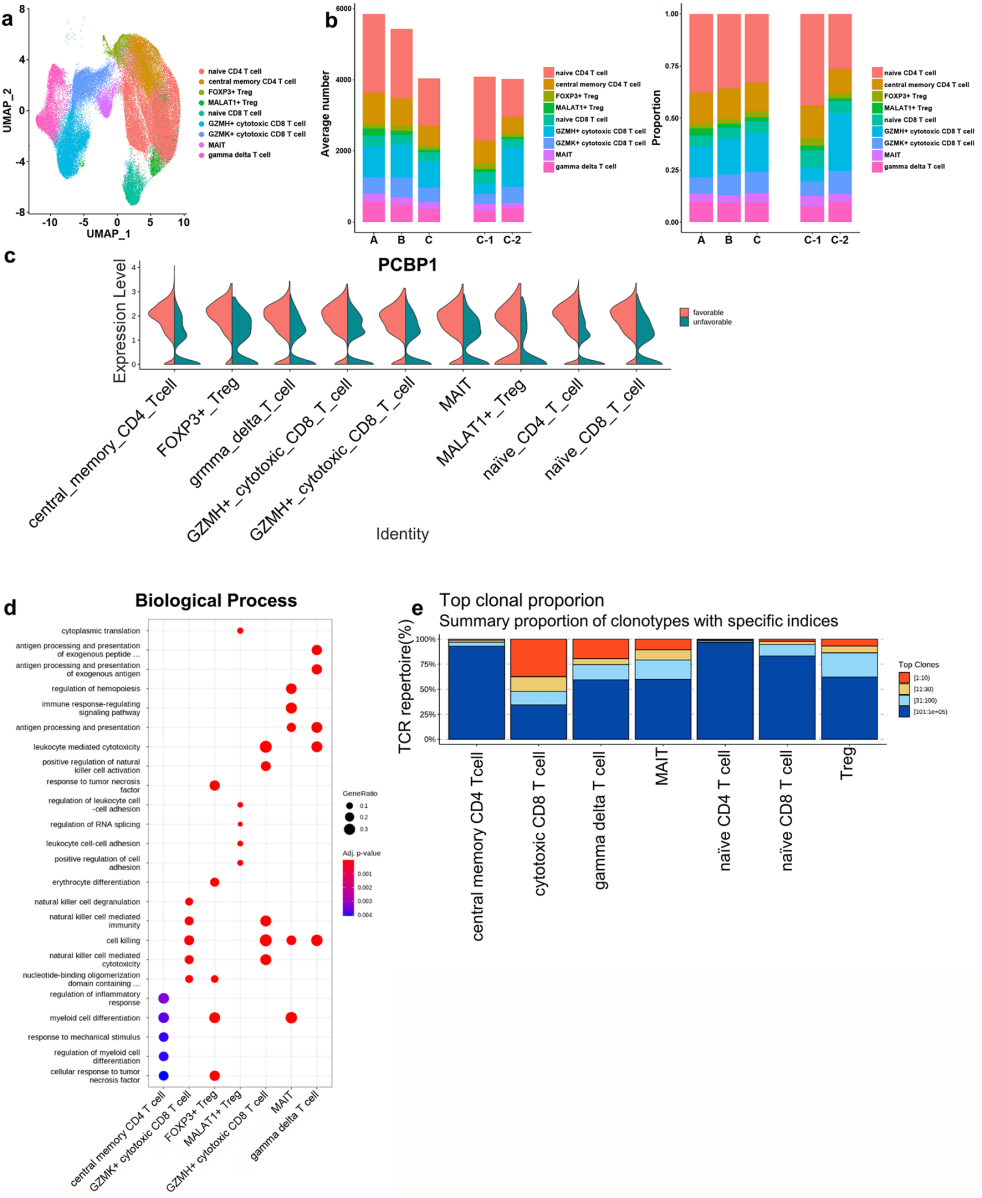
T cell subsets. (**a**) UMAP Plots of T cells color-coded by cell subsets as indicated. MAIT, mucosal-associated invariant T cell. (**b**) Average cell number and relative proportion of T cell subsets from PBMCs of each group. (**c**) Violin plot of PCBP1 expression in each T cell subset by subgroup. (**d**) Functional enrichment analysis of DEGs in subgroup C-1 compared to subgroup C-2. Dot plot showing the most significant GO terms in each cell type. The top 5 GO terms were selected from each cluster. Dot size represents the gene ratio included in each GO term. Color gradient of the dots represents the adjusted p-values of each enriched GO term. (**e**) Distribution of the TCR clonotype frequency by cell type.

Subclustering of T cells revealed 15 clusters converting to seven differentiation states, including naïve CD4^+^ T cells, central memory CD4^+^ T cells, regulatory T cells (Tregs), naïve CD8^+^ T cells, cytotoxic CD8^+^ T cells, mucosal-associated invariant T cells (MAIT), and γδ T cells with distinct markers (Fig. 3a, Supplementary Fig. S3a–c online). There was no difference in the proportion of T cell subsets in the comparison of group C and combined groups A and B (Fig. 3b, Supplementary Fig. S3d,e online, and Supplementary Table S4 online). DEG comparison showed that PCBP1 was highly expressed in subgroup C-1 compared to C-2 (Fig. 3c). DEGs upregulated in C-1 versus C-2 were associated with cell killing, antigen processing and presentation, leukocyte and NK cell-mediated cytotoxicity, response to tumor necrosis factor (TNF), and myeloid cell differentiation (Fig. 3d).

We next performed single-cell T cell receptor sequencing (scTCR-seq) on T cells. By interrogating the paired TCRα and TCRβ repertories in all T cells (n = 104,719), we showed that 81.4% (85,257/104,726) contained at least one productive V-J spanning pair (Supplementary Fig. S4a online). The total number of clonotypes was 80,328. Highly expanded clonal T cells colocalized with cytotoxic CD8+ T cells (Fig. 3e and Supplementary Fig. S4b online). We observed numerous highly expanded clones in groups B and C (Supplementary Fig. S4c,d online). And T cells from each sample harbored a unique clonotype (Supplementary Fig. S4e online). Our paired scRNA and scTCR profiling supported an adaptive T cell repertoire that includes expanded cytotoxic CD8^+^ T cell clonotypes that emerge mainly in MAC-PD patients following disease progression.

### Analysis of NK cell subsets

**Figure 4:**
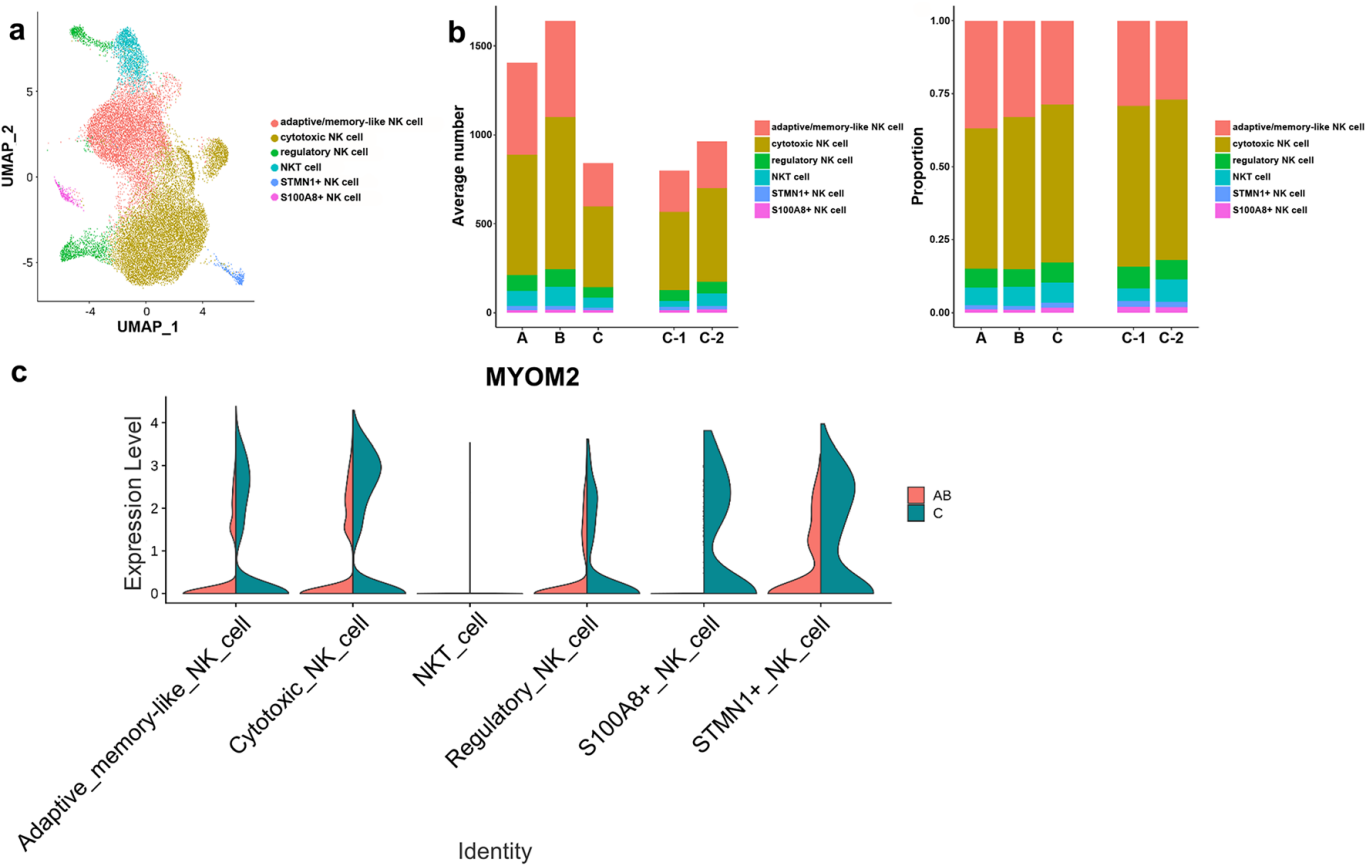
NK cell subsets. (**a**) UMAP plot of NK cells color-coded by cell subsets as indicated. (**b**) Average cell number and relative proportion of NK cell subsets from PBMCs of each group. (**c**) Violin plot of MYOM2 expression in each NK cell subset by group.

Subclustering of NK cells revealed 12 clusters converting to six differentiation states, including regulatory NK cells, cytotoxic NK cells, adaptive/memory-like NK cells, NKT cells, STMN1^+^ NK cells, and S100A8^+^ NK cells with distinct markers (Fig. 4 and Supplementary Fig. S5a–f online). NK subsets also expressed higher MYOM2 levels in group C compared to combined groups A and B (Fig. 4c). GO analysis of upregulated DEGs of NK cell subsets in group C showed significant increases in negative regulation of myeloid cell differentiation, cellular response to TNF, and cellular response to molecule of bacterial origin (Supplementary Fig. S5g online). Also, DEG comparison showed that FOS and KLF6 were highly expressed in subgroup C-1 compared to C-2 among NK cell subtypes (Supplementary Fig. S5h online). GO analysis of upregulated DEGs of NK cell subsets in subgroup C-1 revealed significant increases in antigen processing and presentation (Supplementary Fig. S5i online).

### Analysis of monocyte subsets

**Figure 5:**
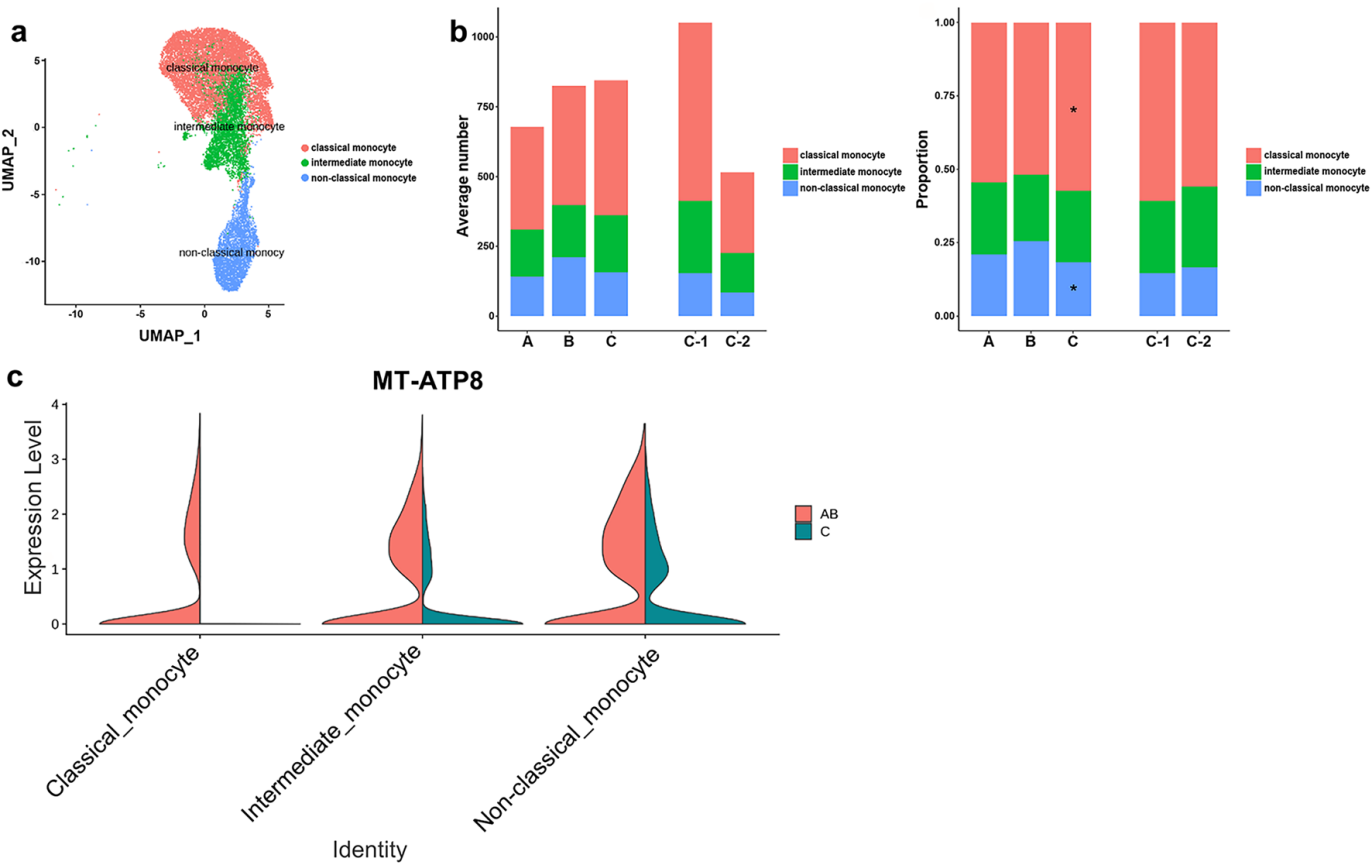
Monocyte subsets. (**a**) UMAP plot of monocytes, color-coded by cell subsets as indicated. (**b**) Average cell number and relative proportion of monocyte subsets from PBMCs of each group. (**c**) Violin plot of MT-ATP8 expression in each monocyte subset by group.

Subclustering of monocytes revealed 14 clusters converting to three differentiation states, including classical monocytes, intermediate monocytes, and non-classical monocytes with distinct markers (Fig. 5a and Supplementary Fig. S6a–d online). In group C compared to groups A and B, the proportion of classical monocytes increased significantly, while the proportion of non-classical monocytes decreased significantly (Fig. 5b, Supplementary Fig. S6e,f online, and Supplementary Tables S3 and S4 online). MT-ATP8 was down-regulated in group C compared to combined groups A and B (Fig. 5c). GO analysis of upregulated DEGs of monocyte subsets in subgroup C-1 revealed significant upregulation in cell chemotaxis and positive regulation of cell adhesion (Supplementary Fig. S6g online).

### Analysis of B cell subsets

**Figure 6:**
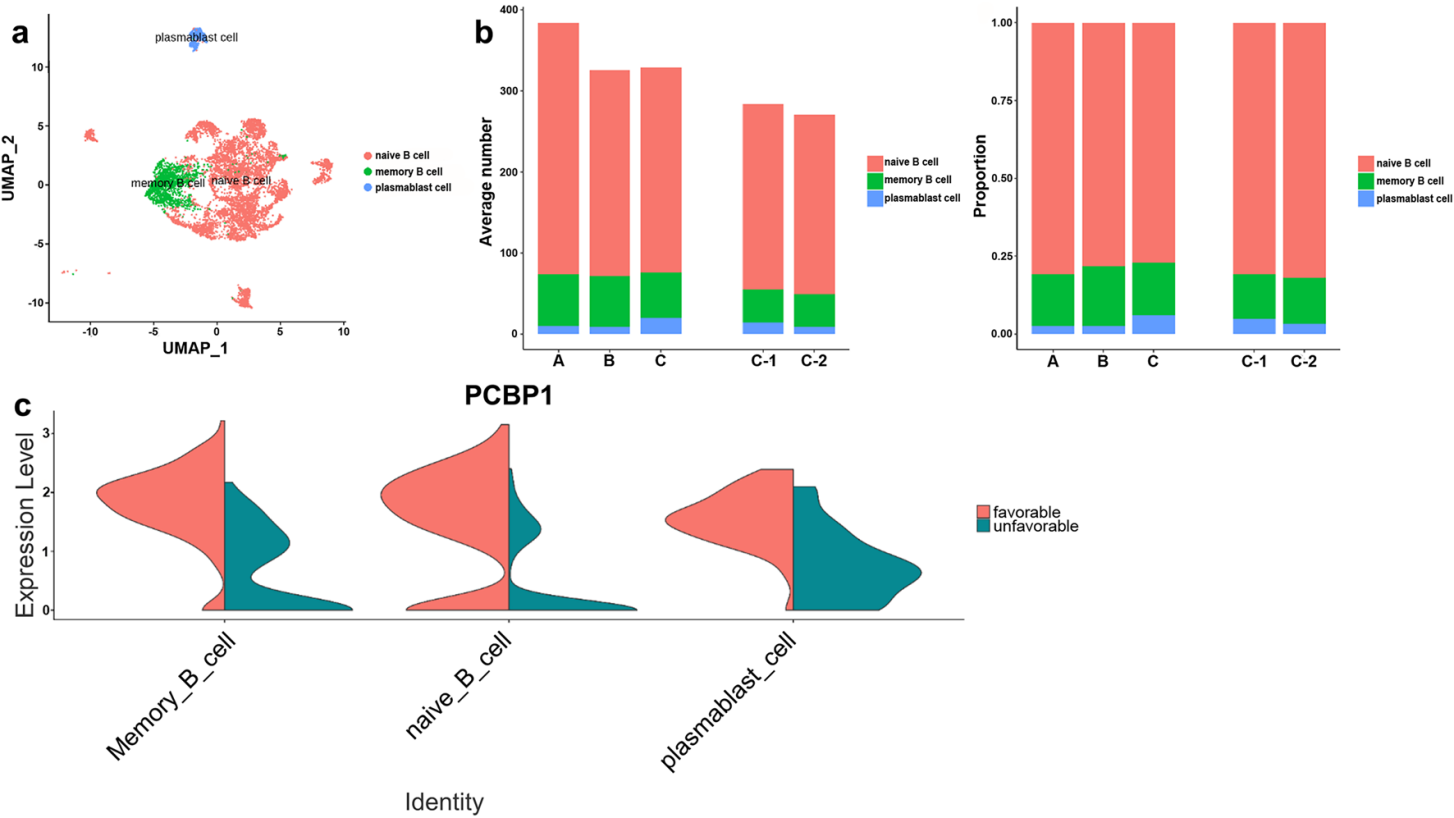
B cell subsets. (**a**) UMAP plots of B cells color-coded by cell subsets as indicated. (**b**) Average cell number and relative proportion of B cell subsets from PBMCs of each group. (**c**) Violin plot of PCBP1 expression in each B cell subset by subgroup.

Subclustering of B cells revealed 18 clusters converting to three differentiation states, including naïve B cells, memory B cells, and plasmablast cells with distinct markers (Fig. 6a and Supplementary Fig. S7a–c online). Among these, naïve B cells were the most abundant in PBMCs (Fig. 6b and Supplementary Fig. S7e,f online). Each B cell subtype displayed immunoglobulin (Ig) encoded by the Ig light/heavy chain variable region gene (IGLV/IGHV) expression profile (Supplementary Fig. S7d online), suggesting the generation and clonal expansion of MAC antigen-specific B cells. The proportion of IGHV5-51^+^ B cells increased significantly in group C compared to combined groups A and B, while proportions of other B cells showed no difference (Supplementary Table S4 online). In the no-treatment group compared to treatment group, CD83 was highly expressed in memory B cells (Supplementary Fig. S7g online). PCBP1 was highly expressed in subgroup C-1 compared to C-2 among B cell subtypes (Fig. 6c).

### Analysis of DC subsets

**Figure 7:**
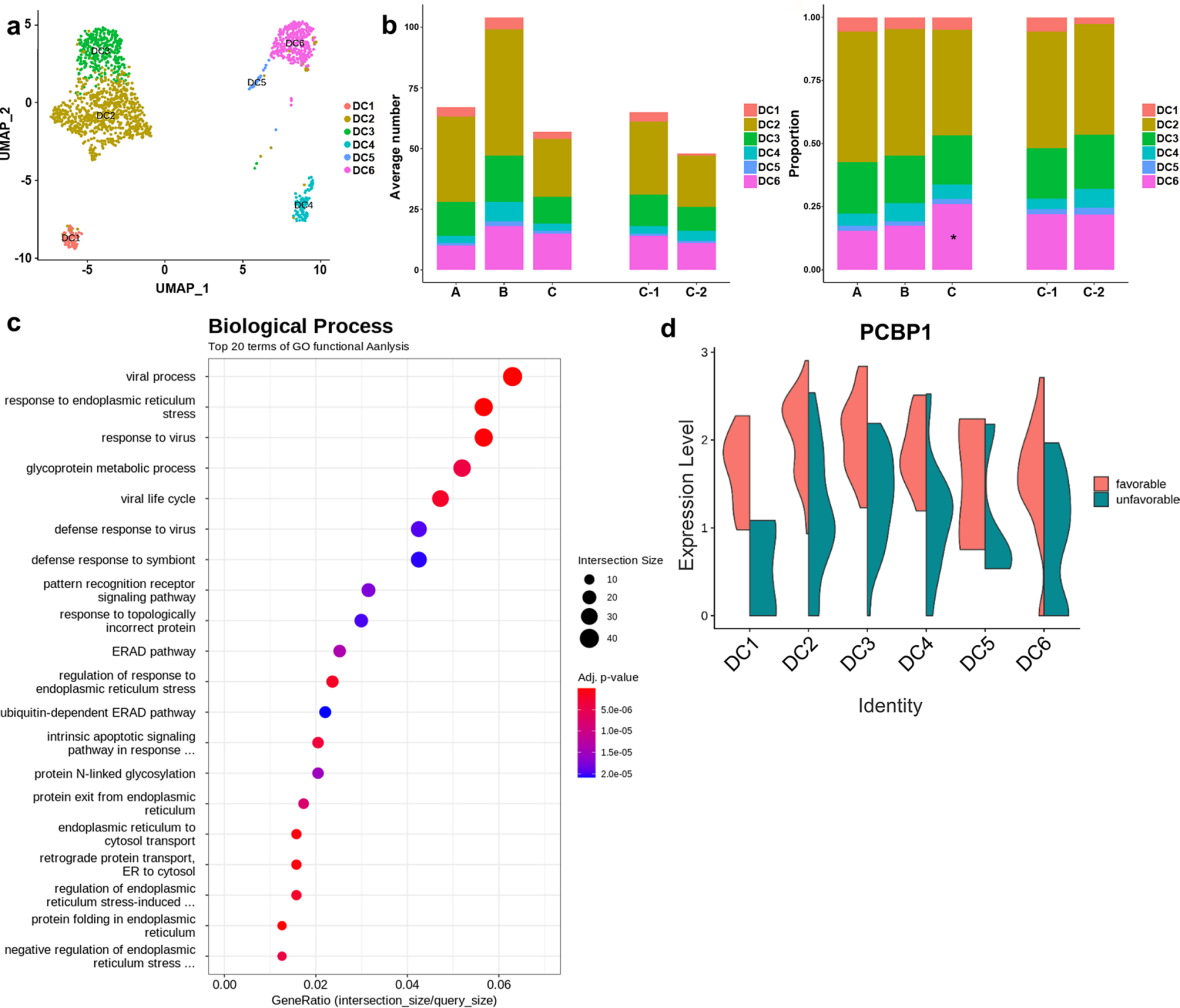
DC subsets. (**a**) UMAP plot of DCs, color-coded by cell subsets as indicated. (**b**) Average cell number and relative proportion of DC subsets from PBMCs of each group. (**c**) GO analysis of DEGs in DC6. (**d**) Violin plot of PCBP1 expression in DC subsets by subgroup.

Subclustering of DCs revealed eight clusters converting to six differentiation states, including CD141^+^ DCs (DC1); CD1c^+^ A DCs (Langerhans cells, DC2); CD163^+^ CD14^+^ DCs (CD1c^+^ B DCs, DC3); CD1c^−^ CD141^−^ CD11c^+^ DCs (DC4); AXL^+^ SIGLEC6^+^ DCs (DC5); and plasmacytoid DCs (pDCs, DC6) with distinct markers (Fig. 7a and Supplementary Fig. S8a–c online). Among DC subsets, the proportion of DC6 increased in group C compared to groups A and B (Fig. 7b, Supplementary Fig. S8d,e online, and Supplementary Tables S3 and S4 online). GO analysis of DEGS in DC6 compared to other DC subsets indicated significant increase in response to ER stress (Fig. 7c). Also, PCBP1 was highly expressed in subgroup C-1 compared to C-2 among DC subtypes (Fig. 7d).

## Discussion

We found decrease in NK cells in patients with progressive disease compared to patients with spontaneous culture conversion or stable disease. In tuberculosis, depletion of a cytotoxic NK subset with high levels of MYOM2 was reported^22^. NK cells primarily produce Th1-type cytokines when responding to intracellular pathogens, including IFN-γ and TNF, which facilitate activation of T cells, DCs, macrophages, and neutrophils^23^. NK cells also produce chemokines that attract effector lymphocytes and myeloid cells to inflamed tissues, and NK cell activation receptors initiate inflammatory transcriptional programs through AP-1 and NF-κB^23^. We propose that the low proportion of NK cells in peripheral blood could be used as a novel factor for distinguishing patients with progressive disease that requires antibiotic administration.

We also observed high proportions of monocytes in the disease progression group. The proportion of classical monocytes was increased in patients with progressive disease compared to patients with spontaneous culture conversion or stable disease, while the proportion of non-classical monocytes decreased. Classical monocytes are highly phagocytic and important scavenger cells. Non-classical monocytes are responsible for CD4^+^ T cell proliferation and stimulation and are involved in antigen presentation. Reduced non-classical monocytes would have resulted in defects in antigen presentation and T cell activation. In disease progression group, the ratio of classical to non-classical monocytes was 3.5 (56% vs. 16%). In spontaneous conversion or stable disease groups, the ratio of those was 1.9 (50% vs. 27%). Based on our results, the balance between classical/non-classical monocyte may be a determinant of disease progression in MAC-PD.

For patients with favorable and unfavorable response to treatment, there was no differences in proportions of five major cell types and subtypes at the pre-treatment time point (Supplementary Table S5 online). Therefore, cell populations may be related to disease progression rather than treatment outcome.

We found several candidates for prediction of the MAC-PD clinical course. HSPA1B was up-regulated in spontaneous conversion group. HSP70, encoded by HSPA1B, is essential for antigen presentation and immune response, potentially via engaging TLR-2/4 receptors, binding to antigenic peptides, and delivering them to antigen-presenting cells (APCs)^24^. HSP70 could inhibit oncogene induction. High HSPA1B expression in T cells may exert antitumor effects in hepatocellulare carcinoma^25^. Also, HSPA1B was significantly down-regulated in the tumor group compared to normal group^26^. Therefore, upregulation of HSPA1B may have contributed to spontaneous culture conversion. MYOM2 in NK cells was specifically up-regulated in disease progression group. MYOM2 is a type of muscle fiber related protein and is an essential component of cytoskeleton. It is also the main component of the M-bands, which are located in the center of the sarcomere and are essential for the stability of sarcomere contraction^27^. In Alzheimer’s disease patients, NK cells were reduced, and a unique NK cell subset expressing MYOM2 was expanded^28^. MYOM2 involved in the process of the hepatocellulare carcinoma prognosis and immune infiltration^29^. And overexpressed MYOM2 was associated with poor prognosis in osteosarcoma^30^. MYOM2 was the only significantly up-regulated gene in localized invasive periodontitis, suggesting that it was associated with inflammation^31^. Our findings suggest that upregulated MYOM2 could be a marker of progressive disease for MAC-PD. In contrast, MT-ATP8 and CD83 were down-regulated in progressive disease group. Down-regulated MT-ATP8 was associated with diabetic kidney disease^32^; additionally, decreased levels of ATPase 8, encoded by MT-ATP8, were associated with rapidly progressive infantile cardiomyopathy^33^. CD83 is expressed on activated B cells and promotes upregulation of MHC-II and CD86 on activated APCs required for T cell activation^34^. Defects in antigen-presenting function may be involved in MAC-PD progression. PCBP1 was up-regulated in group with favorable treatment response. PCBP1 acts as a tumor suppressor gene, inhibiting lung adenocarcinoma development^35^. Lung adenocarcinoma patients with high PCBP1 expression exhibited longer survival, suggesting that PCBP1 may be a marker of good prognosis. PCBP1 may also have prognostic utility as a marker for favorable treatment response in MAC-PD. These candidates based on our scRNA-seq data could not be validated due to the lack of other scRNA-seq data from MAC-PD patients. Further scRNA-seq study in bronchiectasis patients with NTM infection is underway, and comparative results will be presented in the future.

This study had several limitations. First, the baseline characteristics of the patients may be a confounding factor for our results. However, due to the small number of subjects, we were unable to detect and correct for potential confounding effects. Therefore, our preliminary findings should be tested in a larger number of MAC-PD patients for generalization. Second, small sample numbers may result in variation, which limits the statistical power of the data to draw clear conclusions. Third, because it was conducted in a single referral center, our data may not be generalizable to other regions.

To the best of our knowledge, this is the first study to perform scRNA-seq to investigate heterogeneity of immune cell populations among MAC-PD patients with diverse clinical courses. Our findings may offer a comprehensive understanding of the host factors that influence a particular MAC-PD clinical course and could suggest an immunological mechanism associated with the disease progression of MAC-PD.

## Materials and methods

### Study population

The clinical data and study samples were obtained from individuals in the NTM Registry of Samsung Medical Center (ClinicalTrials.gov: NCT00970801), which was approved by the Institutional Review Board (IRB) of Samsung Medical Center (no. 2008-09-016, date of registration: January 2008). The Registry is an ongoing research project aimed at analyzing clinical factors and human-derived materials, including sputum and NTM strains, for the investigation of the pathophysiology of NTM-PD patients. The IRB approval has been renewed annually (IRB no. 2008-09-016-041, latest update on November 3, 2023). All study subjects provided written informed consent. This study was conducted in accordance with the principles of the Declaration of Helsinki.

In this study, 21 patients with MAC-PD were identified (Supplementary Table S1 online). In our hospital, cultures of the respiratory specimens were obtained using standard methods^36^. The processed specimens were inoculated into the BACTEC MGIT system (BD Diagnostics, Sparks, MD, USA). Liquid culture was used for NTM identification. The MAC clinical isolates were identified as *M. avium* or *M. intracellulare* using a commercial diagnostic kit based on the PCR-reverse blot hybridization assay (REBA) of the *rpoB* gene (REBA Myco-ID^®^, YD Diagnostics, Yongin, South Korea) in routine clinical practice^37^. Patients were divided into three categories by the clinical disease course: group A, spontaneous culture conversion; group B, stable disease without antibiotic treatment; and group C, progressive disease with antibiotic treatment. Five patients in group A experienced spontaneous negative culture conversion without antibiotic therapy, which was defined as three consecutive negative cultures of sputum collected at least 4 weeks apart. They also had mild symptoms with radiological lesions that either spontaneously improved or remained stable. Group B comprised seven patients who had stable disease status without any antibiotic treatment. In group B patients, the disease did not progress several years after the diagnosis of MAC-PD. They remained in a state where MAC strains were chronically detected in sputum without exacerbation of respiratory symptoms or worsening on chest radiological examination. The nine patients in group C had progressive disease that required antibiotic administration. Patients in group C experienced exacerbation of symptoms such as cough, sputum production, and fever after the diagnosis of MAC-PD. Additionally, they developed or worsened radiological lesions such as cavity, bronchiolitis, or consolidation on chest radiological examination. These patients started antibiotic therapy. Group C patients were further divided into three subgroups, based on treatment responses; three achieved negative culture conversion after starting antibiotics (favorable response, subgroup C-1), three failed culture conversion despite at least 6 months of antibiotics (unfavorable response, subgroup C-2), while the other three patients could not be classified due to refusal of antibiotic treatment or follow-up loss (subgroup C-x).

### Sample preparation

Peripheral blood from patients was drawn between June 2020 and February 2021, prior to initiation of treatment. PBMCs were isolated via standard Ficoll-Paque (GE Healthcare, Uppsala, Sweden) density gradient centrifugation. All samples showed high viability, about 94% on average. LUNA-FL™ Automated Fluorescence Cell Counter (Logos Biosystems, Anyang, South Korea) was consult the 10× Genomics single cell protocols.

### Library construction and sequencing

scRNA-seq was performed by Macrogen Inc. (Seoul, South Korea). The 5ʹ gene expression libraries and V(D)J enriched libraries were prepared using the Chromium controller according to the 10× Chromium Next GEM Single Cell V(D)J User Guide (10× Genomics, Pleasanton, CA, USA). Briefly, the cell suspensions were diluted in nuclease-free water to achieve a targeted cell count of 10,000. Cell suspension was mixed with master mix and loaded with Single Cell 5′ Gel Beads and Partitioning Oil into a Next GEM Chip G. RNA transcripts from single cells were uniquely barcoded and reverse-transcribed within droplets. cDNA molecules were pooled and enriched with PCR. For V(D)J Enriched Library, the enriched cDNA pool was first amplified using T Cell Mix1 primer and second amplified using T Cell Mix2 primer. For 5ʹ Gene Expression Library, the cDNA pool went through an end repair process, the addition of a single ‘A’ base, and then ligation of the adapters. The products were then purified and enriched with PCR to create the 5’ Gene Expression Library. The libraries were quantified using qPCR according to the qPCR Quantification Protocol Guide (KAPA) and qualified using the Agilent Technologies 4200 TapeStation (Agilent technologies, Santa Clara, CA, USA). And then the libraries were sequenced using HiSeq platform (Illumina, San Diego, CA, USA) according to the read length in the user guide.

### Preprocessing and analysis of scRNA-seq data

We used Cell Ranger v5.0.0 (10× Genomics, Pleasanton, CA, USA) pipeline for FASTQ file generation from raw sequencing data, gene expression analysis of 5ʹ gene expression library data, and immune profiling analysis of V(D)J enriched library data. Illumina basecall files from Illumina sequencing instrument were converted to FASTQ format for each sample using the ‘cellranger mkfastq’ command.

Gene expression libraries were analyzed with the ‘cellranger count’ command. Sequencing reads were aligned to the *GRCh38-2020-A*, genome reference using STAR (v2.5.1b) aligner and assembled for each cell according to the unique molecular identifier (UMI) and 10× cell barcode. Then, cells were grouped into clusters by their gene expression. The count analysis for each sample was aggregated using the ‘cellranger aggr’.

V(D)J enriched libraries were analyzed with the ‘cellranger vdj’ command. Sequencing reads were assembled for each cell according to the UMI and 10× cell barcode. Clonotype calling was performed by matching assembled reads to the IMGT DB (GRCh38-alts-ensembl-5.0.0). The vdj results for each sample were aggregated using the ‘cellranger aggr’.

### Advanced analysis of scRNA-seq data

Raw count matrices of 10× Genomics resulting by Cell Ranger were imported to Seurat 3.1.3. Raw data of 36,601 genes, 198,132 cells were analyzed (Supplementary Table S2 online). Raw count with all genes expressed in ≥ 3 cells and all cells with at least 200 detected genes were used in downstream analysis. First filtered data was a feature-barcode matrix with of 25,005 genes, 197,912 cells after filtering. Since there may be a rare subset of cells with a clear outlier number of genes detected as potential multiplets, and there are any low-quality or dying cells, we filtered out cells with the number of genes over 5000 or mitochondrial counts over 10%. After second filtering, selected count value of 25,005 genes across 189,779 cells was used.

Cell pairwise anchor correspondences between different single-cell data sets were identified with 15-dimensional spaces by Reciprocal PCA^38^. Using these anchors, all datasets were integrated and transformed into a shared space. Gene expression values were scaled and normalized for each gene across all integrated cells. Batch effects were corrected by Reciprocal PCA of Seurat R library when aggregating multiple data sets. Dimensionality reduction of the integrated data was conducted using UMAP^39^. Cell type classification was performed using the SingleR (version 1.0) package based on five combined reference datasets^40^. We annotated each cell by identifying marker genes from reference and using them to compute assignment scores (based on the Spearman correlation across markers) for each cell in the test dataset against each label in the reference. The label with the highest score is the assigned to the test cell. The result of cell type classification was represented with plotScoreHeatmap and DimPlot. Clustering and UMAP analysis were performed based on the statistically significant principal components. Then significant top cluster markers for every cluster compared to all remains cells were determined using minimal fraction of min.pct and Wilcox rank sum test. Additionally, we also compared pairwise groups (A, B and C) within each cluster to identify DEGs between two groups using Wilcox rank sum test. Gene-enrichment and functional annotation analysis for significant gene list from two different results was performed respectively using g:Profiler tool (|fold change|≥ 1.5) ^41^. To visualize top clonal proportion across all samples or cell types using CDR3 AA sequence for clonotype calling, immunarch 0.9.0 package was used^42^.

Seurat analysis data of 10× Genomics scRNA-seq are available in online data supplement (Supplementary information_2). The codes generated during this study are available in online data supplement (Supplementary information_3).

### Statistical analysis

The data are presented as the medians and interquartile ranges. We made three comparisons for proportion of cell type: (1) group A versus B versus C, (2) no treatment group (combined both group A and B) versus treatment group C, (3) subgroup C-1 versus C-2. Comparison for (1) evaluated with the Kruskal–wallis test and comparisons for (2) and (3) evaluated with the Wilcoxon rank sum test. A *p*-value is corrected by Bonferroni’s method. Adjusted *p*-values are reported as *q*-values. A *p*-value < 0.05 was considered significant. Statistical analysis was executed using SAS version 9.4 (SAS Institute Inc., Cary, NC, USA).

### Supplementary Information

Supplementary Information 1.

Supplementary Information 2.

Supplementary Information 3.

## Data Availability

Raw data and processed data of scRNA-seq and scTCR-seq have been deposited in Gene Expression Omnibus with accession number GSE226473.
