# Supplementary material for: Single-cell transcriptomics by clinical course of Mycobacterium avium complex pulmonary disease
Source: Sci Rep. 2024 Jul 8;14:15663. doi: 10.1038/s41598-024-66523-x (PMC11231222; doi:10.1038/s41598-024-66523-x)
Supplement: Supplementary file 1 — Supplementary Information 1. [file 41598_2024_66523_MOESM1_ESM.pdf]

[Supplementary data]

Single-cell transcriptomics by clinical course of *Mycobacterium avium* complex pulmonary disease

Su-Young Kim, Sungmin Zo, Dae Hun Kim, Sung Jae Shin, Byung Woo Jhun

Supplementary Tables

Table S1. Clinical feature of the patients with MAC-PD

Patients No.	Batch No.	Group	Age	Sex	Etiology	Disease type	Cavity	Culture conversion	Disease progression	Initiation of treatment	Treatment response	BMI	Smoking	TB	CPA	BE
14	1	B	68	F	*M. intracellulare*	NB form		Persistant positive	Stable			21.32	never			Yes
8	2	B	69	M	*M. avium*	NB form		Persistant positive	Stable			23.43	ex-smoker			Yes
12	3	B	71	M	*M. intracellulare*	NB form		Persistant positive	Stable			22.63	ex-smoker	Yes		Yes
19	4	A	64	F	*M. avium*	NB form		Spontaneous negative				22.66	never			Yes
20	5	A	51	F	*M. intracellulare*	NB form		Spontaneous negative				21.19	never	Yes		Yes
6	6	C	68	F	*M. intracellulare*	NB form		Persistant positive	Rapid	refusal		19.35	never			Yes
16	7	B	60	F	*M. avium, M. intracellulare*	NB form		Persistant positive	Stable			22.55	never			Yes
13	8	B	52	F	*M. avium*	NB form		Persistant positive	Stable			21.22	never			Yes
17	9	B	75	F	*M. avium*	NB form		Persistant positive	Stable			20.88	never			Yes
7	10	C	45	F	*M. avium*	NB form	Yes	Persistant positive	Rapid	Yes	Favorable response	20.87	never			Yes
10	11	A	53	F	*M. avium*	NB form	Yes	Spontaneous negative				20.42	never			Yes
18	12	C	46	F	*M. avium*	NB form		Persistant positive	Rapid	Yes	Unfavorable response	18.62	never			Yes
9	13	B	50	F	*M. avium*	NB form		Persistant positive	Stable			23.71	never			Yes
15	14	A	59	M	*M. intracellulare*	NB form		Spontaneous negative				22.8	ex-smoker			Yes
5	15	C	54	F	*M. avium*	NB form		Persistant positive	Rapid	refusal		21.03	never			Yes
21	16	A	60	F	*M. avium*	NB form		Spontaneous negative				21.52	never			Yes
4	17	C	55	M	*M. avium*	NB form		Persistant positive	Rapid	Yes	Favorable response	20.56	ex-smoker			Yes
2	18	C	68	F	*M. intracellulare*	FC form	Yes	Persistant positive	Rapid	Yes	Unfavorable response	15.81	never	Yes	Yes	
3	19	C	68	F	*M. intracellulare*	NB form	Yes	Persistant positive	Rapid	Yes	follow-up loss	20.5	never			Yes
1	20	C	60	F	*M. intracellulare*	NB form		Persistant positive	Rapid	Yes	Unfavorable response	20.44	never			Yes
11	21	C	44	F	*M. avium*	NB form		Persistant positive	Rapid	Yes	Favorable response	19.9	never			Yes

NB, nodular bronciectatic; FC, fibrocavitary; BMI, body mass index; TB, tuberculosis; CPA, chronic pulmonary aspergillosis; BE, bronchiectasis.

Table S2. Summary of scRNA-seq data

Group	Experimental batch	Estimated no. of cells (raw data)	No. of single-cells (filtered data)
B	1	11,622	10,383
B	2	9,424	9,369
B	3	10,353	10,239
A	4	13,754	12,365
A	5	13,173	12,726
C	6	7,409	6,932
B	7	8,774	8,448
B	8	11,082	10,908
B	9	14,169	13,379
C	10	6,274	6,201
A	11	8,976	8,741
C	12	5,889	5,718
B	13	7,867	7,813
A	14	6,009	5,275
C	15	7,656	7,258
A	16	13,597	13,195
C	17	8,851	8,672
C	18	8,022	7,651
C	19	8,356	8,164
C	20	7,950	7,696
C	21	8,925	8,646
Total		198,132	189,779

Table S3. Proportion of cell types in the three groups A, B, and C

Cell type	Total (n=21)	A (n=5)	B (n=7)	C (n=9)	*p*-value	*q*-value
Total cell						
T	65.29 (58.71, 68.73)	67.56 (65.29, 68.73)	60.52 (55.37, 73.78)	64.88 (58.44, 67.31)	0.5568	1.0000
NK	15.64 (12.45, 22.81)	18.83 (14.08, 24.6)	22.81 (14.86, 24.54)	13.1 (11.93, 15.64)	0.1002	0.5010
Monocyte	12.18 (8.66, 15.66)	8.66 (6.19, 10.53)	12.18 (7.46, 15.47)	15.66 (12.13, 19.45)	0.0703	0.3515
B	5.15 (3.88, 6.96)	5.05 (3.93, 6.02)	3.88 (2.6, 5.15)	7.78 (6.03, 8.89)	0.05	0.2500
DC	1.13 (0.74, 1.22)	0.61 (0.6, 0.8)	1.15 (1.12, 1.39)	1.14 (0.74, 1.19)	0.1159	0.5795
**T cell subtype**						
gamma delta T	8.43 (6.72, 10.5)	8.43 (7.28, 9.79)	6.72 (5.45, 9.83)	10.4 (7.58, 10.79)	0.2789	1.0000
naïve CD8	5.72 (4.43, 7.8)	6.45 (3.09, 7.16)	5.27 (2.53, 7.16)	7.19 (5.01, 7.88)	0.5983	1.0000
cytotoxic CD8	24.88 (18.14, 32.11)	20.52 (18.14, 26.5)	26.88 (13.15, 39.31)	24.88 (21.57, 30.72)	0.8033	1.0000
MAIT	3.79 (3.05, 4.85)	3.75 (3.11, 4.85)	3.3 (2.95, 4.57)	4.02 (2.77, 5.58)	0.8922	1.0000
naïve CD4 T	36.28 (28.83, 41.43)	37.02 (36.28, 41)	34.68 (27.01, 48.73)	33.73 (28.83, 39.39)	0.7496	1.0000
central memory CD4 T	14.69 (12.01, 16.35)	13.21 (13.16, 15.01)	14.69 (11.04, 17.31)	14.69 (11.37, 15.5)	0.8999	1.0000
Treg	4.73 (4.03, 5.75)	5.68 (5.36, 6.29)	4.57 (3.54, 5.21)	4.3 (4.03, 5.76)	0.1928	1.0000
GZMH cytotoxic CD8	61.63 (58.04, 68.03)	65.92 (62.73, 68.03)	61.63 (57.82, 70.46)	60.71 (57.47, 62.81)	0.5413	1.0000
GZMK cytotoxic CD8	38.37 (31.97, 41.96)	34.08 (31.97, 37.27)	38.37 (29.54, 42.18)	39.29 (37.19, 42.53)	0.5413	1.0000
FOXP3 Treg	57.37 (44.09, 69.61)	41.91 (41.35, 42.2)	57.37 (50, 69.61)	64.49 (55.16, 71.51)	0.07	1.0000
MALAT1 Treg	42.63 (30.39, 55.91)	58.09 (57.8, 58.65)	42.63 (30.39, 50)	35.51 (28.49, 44.84)	0.07	1.0000
**NK cell subtype**						
adaptive memory like NK	29.26 (27.02, 40.01)	40.01 (28.97, 42.98)	30.92 (28.13, 43.46)	28.32 (25.89, 29.84)	0.2655	1.0000
cytotoxic NK	54.67 (44, 57.47)	44.19 (41.4, 54.67)	50.53 (44, 56.29)	56.23 (51.63, 59.01)	0.568	1.0000
NKT	5.8 (4.25, 7.81)	4.48 (4.25, 6.41)	5.8 (3.93, 6.57)	7.58 (4.26, 10.35)	0.7776	1.0000
regulatory NK	6.71 (5.46, 7.65)	5.6 (5.46, 8.1)	6.2 (4.8, 6.72)	6.77 (6.71, 7.65)	0.4761	1.0000
S100A8 NK	1.23 (0.81, 1.74)	0.95 (0.81, 1.31)	0.81 (0.75, 1.62)	1.5 (1.23, 2.18)	0.304	1.0000
STMN1 NK	1.3 (1.11, 2.12)	1.3 (0.95, 2.34)	1.15 (1.06, 1.49)	1.41 (1.16, 2.15)	0.3439	1.0000
**monocyte subtype**						
classical monocyte	53.3 (49.45, 57.86)	50.91 (39.26, 53.75)	49.45 (49.03, 53.19)	55.9 (53.94, 60.12)	0.0198	0.2970
intermediate monocyte	24.31 (21.07, 25.99)	24.31 (23.26, 24.38)	21.28 (19.12, 25.99)	25 (23.1, 26.51)	0.6359	1.0000
non classical monocyte	21.88 (15.79, 27.69)	21.88 (21.4, 44.48)	27.69 (23.14, 29.27)	15.79 (14.88, 20.25)	0.0305	0.4575
**B cell subtype**						
naïve B	78.11 (76.8, 81.51)	81.51 (71.24, 82.43)	77.97 (77.62, 79.89)	78.11 (76.8, 81.11)	0.8357	1.0000
memory B	16.38 (15.03, 20.79)	15.91 (14.86, 21.28)	19.58 (16.2, 20.79)	16.16 (15.03, 16.84)	0.5228	1.0000
plasmablast	3.74 (2.12, 6.71)	2.7 (2.58, 4.35)	2.61 (1.69, 3.91)	6.37 (3.74, 7.04)	0.1635	1.0000
IGHV1-18 B	2.52 (1.42, 3.69)	3.42 (2.64, 3.69)	1.82 (0.9, 4.13)	2.52 (2.11, 3.2)	0.6567	1.0000
IGHV1-2 B	5.06 (3.24, 5.6)	4.51 (2.82, 5.54)	5.45 (2.7, 6.61)	5.06 (3.56, 5.35)	0.7392	1.0000
IGHV3- 21 B	6.07 (5.69, 6.96)	6.06 (5.94, 6.07)	6.61 (5.11, 7.69)	6.37 (5.69, 7.33)	0.6677	1.0000
IGHV3-23 B	5.76 (4, 6.52)	6.06 (5.54, 6.15)	5.45 (3.6, 6.52)	5.76 (4, 6.68)	0.6619	1.0000
IGHV3-30 B	8.99 (6.06, 9.96)	6.57 (6.06, 8.88)	7.47 (5.41, 9.09)	9.48 (9.13, 10.97)	0.1403	1.0000
IGHV30-43D B	4.13 (2.96, 5.69)	4.3 (3.03, 4.75)	4.13 (2.1, 5.71)	3.24 (2.59, 6.15)	0.9864	1.0000
IGHV3-53 B	2.25 (1.2, 3.48)	2.25 (1.37, 2.37)	3.48 (1.2, 3.64)	1.93 (1.07, 2.41)	0.3285	1.0000
IGHV5-51 B	2.82 (1.85, 3.31)	1.85 (0.91, 2.82)	2.59 (1.2, 3.31)	3.04 (2.67, 3.6)	0.0935	1.0000
IGKV3-11 B	6.06 (4.57, 7.81)	6.15 (5.74, 6.33)	6.52 (5.79, 7.81)	4.57 (4.32, 8)	0.4397	1.0000
IGKV3-15 B	13.67 (12.59, 16.9)	16.9 (14.55, 17.08)	16.53 (12.59, 18.7)	13.1 (12.36, 13.67)	0.204	1.0000
IGKV3-20 B	10.5 (9.25, 11.71)	10.33 (9.43, 11.08)	11.11 (9.09, 12.17)	10.5 (9.25, 11.39)	0.8324	1.0000
IGLC3 B	20.86 (19.6, 21.72)	21.13 (19.26, 21.72)	20.4 (19.52, 24.48)	20.97 (19.89, 21.71)	0.8368	1.0000
IGLV2-14 B	7.87 (6.61, 9.43)	8.88 (7.12, 9.43)	6.61 (6.31, 10.06)	7.87 (7.59, 8.63)	0.7878	1.0000
IGLV3-1 B	2.28 (1.85, 2.62)	1.85 (1.41, 2.51)	2.1 (0.7, 2.42)	2.53 (2.16, 3.04)	0.1798	1.0000
**DC subtype**						
DC1	5 (2.56, 6.82)	5.68 (3.03, 6.77)	4.29 (2.29, 6.86)	5 (2.56, 6.82)	0.9898	1.0000
DC2	47.24 (42.65, 51.52)	51.52 (43.61, 61.36)	47.24 (41.57, 59.09)	43.64 (37.18, 51.28)	0.2801	1.0000
DC3	19.44 (15.48, 22.14)	19.55 (19.32, 21.21)	18.63 (15.48, 19.7)	19.44 (15, 23.08)	0.8507	1.0000
DC4	5.36 (4.51, 6.94)	5.36 (4.51, 5.68)	5.95 (4.55, 11.04)	5.13 (4.41, 6.94)	0.5437	1.0000
DC5	1.79 (0, 3.01)	1.79 (0, 3.01)	1.23 (0, 3.37)	2.5 (1.39, 2.56)	0.8192	1.0000
DC6	19.44 (16.07, 24.72)	12.12 (7.95, 16.07)	16.67 (16.56, 24.72)	24.59 (19.44, 29.09)	0.0292	0.8760

Table S4. Proportion of cell types in the two groups A+B and C

Cell type	Total (n=21)	A+B (n=12)	C (n=9)	*p*-value	*q*-value
Total cell					
T	65.29 (58.71, 68.73)	66.42 (58.97, 71.26)	64.88 (58.44, 67.31)	0.5458	1.0000
NK	15.64 (12.45, 22.81)	20.82 (14.47, 24.57)	13.1 (11.93, 15.64)	0.036	0.1800
Monocyte	12.18 (8.66, 15.66)	10.83 (6.83, 14.34)	15.66 (12.13, 19.45)	0.0428	0.2140
B	5.15 (3.88, 6.96)	4.22 (2.7, 5.58)	7.78 (6.03, 8.89)	0.0173	0.0865
DC	1.13 (0.74, 1.22)	1.12 (0.7, 1.29)	1.14 (0.74, 1.19)	1.0000	1.0000
**T cell subtype**					
gamma delta T	8.43 (6.72, 10.5)	7.36 (6.5, 9.81)	10.4 (7.58, 10.79)	0.241	1.0000
naïve CD8	5.72 (4.43, 7.8)	5.5 (2.98, 7.16)	7.19 (5.01, 7.88)	0.3744	1.0000
cytotoxic CD8	24.88 (18.14, 32.11)	23.51 (17.11, 33.9)	24.88 (21.57, 30.72)	0.8036	1.0000
MAIT	3.79 (3.05, 4.85)	3.53 (3.08, 4.71)	4.02 (2.77, 5.58)	0.9151	1.0000
naïve CD4 T	36.28 (28.83, 41.43)	36.65 (28.97, 43.27)	33.73 (28.83, 39.39)	0.4996	1.0000
central memory CD4 T	14.69 (12.01, 16.35)	14.32 (12.59, 16.83)	14.69 (11.37, 15.5)	0.7491	1.0000
Treg	4.73 (4.03, 5.75)	4.97 (4.05, 5.71)	4.3 (4.03, 5.76)	0.9151	1.0000
GZMH cytotoxic CD8	61.63 (58.04, 68.03)	64.33 (59.57, 68.58)	60.71 (57.47, 62.81)	0.3374	1.0000
GZMK cytotoxic CD8	38.37 (31.97, 41.96)	35.67 (31.42, 40.43)	39.29 (37.19, 42.53)	0.3374	1.0000
FOXP3 Treg	57.37 (44.09, 69.61)	53.11 (41.63, 63.53)	64.49 (55.16, 71.51)	0.0817	1.0000
MALAT1 Treg	42.63 (30.39, 55.91)	46.89 (36.47, 58.37)	35.51 (28.49, 44.84)	0.0817	1.0000
**NK cell subtype**					
adaptive memory like NK	29.26 (27.02, 40.01)	32.88 (28.4, 43.22)	28.32 (25.89, 29.84)	0.1265	1.0000
cytotoxic NK	54.67 (44, 57.47)	50.48 (42.7, 56.07)	56.23 (51.63, 59.01)	0.3744	1.0000
NKT	5.8 (4.25, 7.81)	5.78 (4.15, 6.49)	7.58 (4.26, 10.35)	0.5458	1.0000
regulatory NK	6.71 (5.46, 7.65)	5.9 (5.31, 7.41)	6.77 (6.71, 7.65)	0.3028	1.0000
S100A8 NK	1.23 (0.81, 1.74)	0.81 (0.8, 1.47)	1.5 (1.23, 2.18)	0.1886	1.0000
STMN1 NK	1.3 (1.11, 2.12)	1.22 (1.03, 1.61)	1.41 (1.16, 2.15)	0.1886	1.0000
**monocyte subtype**					
classical monocyte	53.3 (49.45, 57.86)	50.03 (44.56, 53.47)	55.9 (53.94, 60.12)	0.0062	0.0930
intermediate monocyte	24.31 (21.07, 25.99)	23.78 (19.87, 25.57)	25 (23.1, 26.51)	0.3744	1.0000
non classical monocyte	21.88 (15.79, 27.69)	26.71 (21.64, 31.6)	15.79 (14.88, 20.25)	0.0116	0.1740
**B cell subtype**					
naïve B	78.11 (76.8, 81.51)	78.39 (74.43, 81.97)	78.11 (76.8, 81.11)	0.9151	1.0000
memory B	16.38 (15.03, 20.79)	19.34 (15.39, 21.04)	16.16 (15.03, 16.84)	0.4138	1.0000
plasmablast	3.74 (2.12, 6.71)	2.66 (1.88, 4.13)	6.37 (3.74, 7.04)	0.07	1.0000
IGHV1-18 B	2.52 (1.42, 3.69)	2.52 (1.1, 3.91)	2.52 (2.11, 3.2)	1.0000	1.0000
IGHV1-2 B	5.06 (3.24, 5.6)	5.17 (2.76, 5.88)	5.06 (3.56, 5.35)	0.8036	1.0000
IGHV3- 21 B	6.07 (5.69, 6.96)	6.06 (5.55, 6.78)	6.37 (5.69, 7.33)	0.8589	1.0000
IGHV3-23 B	5.76 (4, 6.52)	5.66 (4.25, 6.33)	5.76 (4, 6.68)	0.6959	1.0000
IGHV3-30 B	8.99 (6.06, 9.96)	7.02 (5.8, 8.99)	9.48 (9.13, 10.97)	0.0597	1.0000
IGHV30-43D B	4.13 (2.96, 5.69)	4.22 (3, 5.55)	3.24 (2.59, 6.15)	0.9717	1.0000
IGHV3-53 B	2.25 (1.2, 3.48)	2.43 (1.28, 3.54)	1.93 (1.07, 2.41)	0.3744	1.0000
IGHV5-51 B	2.82 (1.85, 3.31)	2.22 (1.06, 3.15)	3.04 (2.67, 3.6)	0.0428	1.0000
IGKV3-11 B	6.06 (4.57, 7.81)	6.24 (5.77, 7.4)	4.57 (4.32, 8)	0.3744	1.0000
IGKV3-15 B	13.67 (12.59, 16.9)	16.72 (12.86, 17.91)	13.1 (12.36, 13.67)	0.0817	1.0000
IGKV3-20 B	10.5 (9.25, 11.71)	10.71 (9.26, 11.94)	10.5 (9.25, 11.39)	0.8036	1.0000
IGLC3 B	20.86 (19.6, 21.72)	20.53 (19.39, 23.1)	20.97 (19.89, 21.71)	0.7491	1.0000
IGLV2-14 B	7.87 (6.61, 9.43)	7.91 (6.31, 9.74)	7.87 (7.59, 8.63)	0.8035	1.0000
IGLV3-1 B	2.28 (1.85, 2.62)	1.97 (1, 2.46)	2.53 (2.16, 3.04)	0.0699	1.0000
**DC subtype**					
DC1	5 (2.56, 6.82)	4.99 (2.66, 6.81)	5 (2.56, 6.82)	0.915	1.0000
DC2	47.24 (42.65, 51.52)	48.62 (43.23, 60.23)	43.64 (37.18, 51.28)	0.1657	1.0000
DC3	19.44 (15.48, 22.14)	19.43 (16.73, 20.45)	19.44 (15, 23.08)	0.9151	1.0000
DC4	5.36 (4.51, 6.94)	5.52 (4.53, 7.52)	5.13 (4.41, 6.94)	0.8869	1.0000
DC5	1.79 (0, 3.01)	1.51 (0, 3.19)	2.5 (1.39, 2.56)	0.6378	1.0000
DC6	19.44 (16.07, 24.72)	16.62 (10.04, 21.95)	24.59 (19.44, 29.09)	0.0209	0.6270

Table S5. Proportion of cell types in the two subgroups C-1 and C-2

Cell type	Total (n=6)	C-1 (n=3)	C-2 (n=3)	*p*-value	*q*-value
Total cell					
T	65.23 (60.69, 70.55)	65.58 (48.55, 70.55)	64.88 (60.69, 74.34)	1.0000	1.0000
NK	13.78 (9.04, 20.81)	9.04 (6.36, 22.26)	15.64 (11.93, 20.81)	0.6625	1.0000
Monocyte	14.39 (11.78, 18.69)	18.69 (15.66, 22.03)	11.78 (3.77, 13.11)	0.0809	0.4045
B	7.01 (4.01, 8.89)	6.03 (4.01, 7.99)	8.89 (0.62, 11.04)	0.6625	1.0000
DC	1 (0.74, 1.19)	1.14 (0.74, 1.39)	0.86 (0.46, 1.19)	0.6625	1.0000
**T cell subtype**					
gamma delta T	8.99 (6.44, 10.5)	6.44 (6.3, 10.5)	10.4 (7.58, 12.21)	0.3827	1.0000
naïve CD8	7.84 (5.01, 8.01)	7.88 (7.8, 8.01)	5.01 (4.69, 8.29)	0.6625	1.0000
cytotoxic CD8	23.77 (9.96, 30.72)	9.96 (8.71, 24.88)	30.72 (22.66, 54.64)	0.1904	1.0000
MAIT	4.15 (2.64, 5.58)	5.58 (2.64, 6.42)	3.79 (2.3, 4.5)	0.3827	1.0000
naïve CD4 T	38.84 (30.49, 45.11)	45.11 (39.39, 46.45)	30.49 (17.12, 38.29)	0.0809	1.0000
central memory CD4 T	14.5 (11.37, 15.29)	14.69 (14.31, 17.27)	11.37 (8.48, 15.29)	0.3827	1.0000
Treg	4.59 (4.3, 6.53)	6.53 (4.3, 6.83)	4.3 (2.98, 4.88)	0.1904	1.0000
GZMH cytotoxic CD8	59.38 (57.47, 70.34)	57.47 (32.29, 58.04)	70.34 (60.71, 76.75)	0.0809	1.0000
GZMK cytotoxic CD8	40.63 (29.66, 42.53)	42.53 (41.96, 67.71)	29.66 (23.25, 39.29)	0.0809	1.0000
FOXP3 Treg	67.08 (50.88, 72.37)	64.49 (50.88, 75.48)	69.68 (44.09, 72.37)	1.0000	1.0000
MALAT1 Treg	32.92 (27.63, 49.12)	35.51 (24.52, 49.12)	30.32 (27.63, 55.91)	1.0000	1.0000
**NK cell subtype**					
adaptive memory like NK	28.79 (25.89, 29.84)	29.26 (25.89, 35.06)	28.32 (25.07, 29.84)	0.6625	1.0000
cytotoxic NK	55.7 (51.63, 57.47)	57.47 (36.28, 59.01)	55.17 (51.63, 56.23)	0.6625	1.0000
NKT	6.27 (2.95, 10.35)	2.95 (2, 14.33)	7.58 (4.97, 10.35)	0.6625	1.0000
regulatory NK	6.84 (6.71, 7.65)	6.74 (6.71, 9.71)	6.95 (4.83, 7.65)	1.0000	1.0000
S100A8 NK	1.76 (1.23, 2.44)	2.18 (1.23, 2.44)	1.35 (0.14, 4.56)	1.0000	1.0000
STMN1 NK	2.14 (1.4, 2.16)	2.16 (1.4, 5.18)	2.12 (1.09, 2.15)	0.3827	1.0000
**monocyte subtype**					
classical monocyte	59.28 (53.94, 61.06)	61.06 (58.45, 64.89)	53.94 (53.3, 60.12)	0.1904	1.0000
intermediate monocyte	25.38 (23.99, 27.09)	23.99 (23.1, 25.76)	27.09 (25, 34.8)	0.1904	1.0000
non classical monocyte	14.92 (12.02, 15.79)	14.95 (12.02, 15.79)	14.88 (11.89, 18.97)	1.0000	1.0000
**B cell subtype**					
naïve B	80.5 (78.11, 82.87)	81.11 (78.11, 82.87)	79.89 (64.1, 85.38)	1.0000	1.0000
memory B	15.6 (12.5, 16.38)	15.03 (11.85, 16.16)	16.38 (12.5, 23.08)	0.3827	1.0000
plasmablast	4.73 (2.12, 7.04)	5.72 (2.1, 7.04)	3.74 (2.12, 12.82)	1.0000	1.0000
IGHV1-18 B	2.55 (2.11, 3.2)	2.59 (2.11, 3.2)	2.52 (0, 4.7)	1.0000	1.0000
IGHV1-2 B	5.02 (3.24, 5.6)	5.6 (5.06, 6.39)	3.24 (0, 4.97)	0.0809	1.0000
IGHV3- 21 B	6.86 (5.8, 7.59)	7.33 (6.39, 7.59)	5.8 (4, 8.27)	0.6625	1.0000
IGHV3-23 B	4.76 (3.88, 5.76)	3.88 (3.2, 7.17)	5.52 (4, 5.76)	0.6625	1.0000
IGHV3-30 B	10.23 (9.13, 12.95)	9.48 (9.13, 10.97)	12.95 (0, 13.54)	0.6625	1.0000
IGHV30-43D B	2.89 (2.21, 3.24)	3.2 (2.59, 6.33)	2.21 (0, 3.24)	0.3827	1.0000
IGHV3-53 B	1.65 (0.84, 2.16)	0.84 (0.43, 1.37)	2.16 (1.93, 4)	0.0809	1.0000
IGHV5-51 B	3.52 (3.04, 4)	3.45 (2.11, 4.11)	3.6 (3.04, 4)	1.0000	1.0000
IGKV3-11 B	6.28 (4.32, 8.29)	4.57 (3.8, 9.05)	8 (4.32, 8.29)	1.0000	1.0000
IGKV3-15 B	13.16 (11.64, 15.98)	12.66 (11.64, 15.98)	13.67 (9.94, 16)	1.0000	1.0000
IGKV3-20 B	11.22 (10.5, 11.87)	11.39 (10.5, 12.93)	11.05 (8, 11.87)	0.6625	1.0000
IGLC3 B	20.51 (19.83, 22.83)	21.12 (19.83, 22.83)	19.89 (17.63, 32)	1.0000	1.0000
IGLV2-14 B	7.68 (6.85, 8.63)	7.59 (6.85, 7.76)	8.63 (6.08, 16)	0.6625	1.0000
IGLV3-1 B	2.41 (2.16, 3.04)	2.28 (2.16, 2.53)	3.04 (2.16, 4)	0.3827	1.0000
**DC subtype**					
DC1	3.21 (1.39, 7.35)	7.35 (1.39, 9.09)	2.56 (0, 3.85)	0.3827	1.0000
DC2	47.46 (42.65, 51.39)	43.64 (42.65, 51.39)	51.28 (37.18, 51.72)	1.0000	1.0000
DC3	21.26 (14.55, 23.08)	19.44 (14.55, 25)	23.08 (13.79, 23.08)	1.0000	1.0000
DC4	4.77 (3.45, 6.94)	4.41 (0, 6.94)	5.13 (3.45, 10.26)	0.6625	1.0000
DC5	2.56 (1.47, 3.45)	1.47 (139, 3.64)	2.56 (2.56, 3.45)	0.6579	1.0000
DC6	21.26 (19.12, 27.59)	19.44 (19.12, 29.09)	23.08 (15.38, 27.59)	1.0000	1.0000

Table S6. Proportion of cell types in the three groups A, B, and C of *M. avium* patients

Cell type	A (n=3)	B (n=4)	C (n=5)	A+B (n=7)	A vs B vs C		A+B vs C	
					p-value	*q*-value	*p*-value	*q*-value
**Total cell**								
T	67.56 (66.42, 70.99)	64.4 (59.24, 69.65)	65.58 (60.69, 67.31)	67.56 (62.91, 71.03)	0.5751	1	0.5160	1
NK	14.08 (12.79, 16.46)	19.38 (13.46, 24.05)	12.45 (9.04, 15.64)	14.86 (12.79, 21.36)	0.4496	1	0.3299	1
Monocyte	8.66 (7.42, 11.39)	12.83 (10.21, 14.78)	15.66 (12.13, 18.69)	11.12 (8.06, 14.34)	0.1313	0.6566	0.0740	0.3702
B	6.02 (4.97, 6.37)	3.34 (2.75, 4.6)	6.96 (6.03, 7.99)	3.93 (3.34, 6.37)	0.0912	0.4562	0.0513	0.2566
DC	0.8 (0.65, 1.08)	1.17 (1.12, 1.26)	1.14 (0.86, 1.15)	1.13 (0.95, 1.29)	0.4791	1	1	1
**T cell subtype**								
gamma delta T	9.79 (8.53, 12.18)	8.27 (6.61, 12.65)	8.89 (6.44, 10.5)	9.79 (7, 12.19)	0.8067	1	0.7453	1
naïve CD8	3.09 (2.98, 4.77)	5.5 (4.41, 6.08)	7.88 (7.8, 8.01)	5.27 (2.98, 6.08)	0.0602	1	0.0230	0.8047
cytotoxic CD8	20.52 (19.33, 26.31)	37.5 (33.49, 39.81)	21.57 (9.96, 22.66)	32.11 (23.7, 37.5)	0.0355	1	0.0740	1
MAIT	3.75 (3.43, 4.3)	3.84 (3.08, 4.83)	5.58 (2.64, 6.42)	3.75 (3.12, 4.71)	0.9171	1	0.7453	1
naïve CD4 T	41 (35.8, 41.21)	27.17 (25.18, 29.16)	39.39 (38.29, 45.11)	30.61 (27.17, 37.84)	0.0556	1	0.1044	1
central memory CD4 T	13.16 (12.59, 16.14)	12.86 (10.62, 15.34)	14.69 (14.31, 16.38)	13.16 (11.52, 16)	0.7939	1	0.7453	1
Treg	5.68 (4.93, 5.99)	3.73 (3.53, 4.08)	5.76 (4.88, 6.53)	4.19 (3.73, 5.12)	0.0507	1	0.0740	1
GZMH cytotoxic CD8	65.92 (62.34, 66.97)	61 (59.74, 62.75)	57.47 (56.04, 58.04)	61.63 (59.57, 66.01)	0.2665	1	0.1439	1
GZMK cytotoxic CD8	34.08 (33.03, 37.66)	39 (37.25, 40.26)	42.53 (41.96, 43.96)	38.37 (33.99, 40.43)	0.2665	1	0.1439	1
FOXP3 Treg	42.2 (35.63, 55.17)	64.27 (56.7, 71.78)	55.16 (50.88, 64.49)	58.93 (46.1, 68.88)	0.3029	1	1	1
MALAT1 Treg	57.8 (44.83, 64.37)	35.73 (28.22, 43.3)	44.84 (35.51, 49.12)	41.07 (31.12, 53.9)	0.3029	1	1	1
**NK cell subtype**								
adaptive memory like NK	28.97 (28, 35.98)	29.52 (27.72, 34.05)	28.32 (25.89, 29.26)	28.97 (27.57, 36.95)	0.6707	1	0.4168	1
cytotoxic NK	54.67 (47.79, 56.76)	53.14 (47.51, 55.96)	57.47 (55.17, 59.01)	54.67 (45.66, 56.07)	0.5520	1	0.3299	1
NKT	4.25 (4.15, 5.33)	6.18 (5.79, 8.69)	4.97 (2.95, 13.55)	5.8 (5, 6.49)	0.3794	1	0.6261	1
regulatory NK	8.1 (6.78, 8.34)	6.46 (5.85, 7.08)	6.74 (6.71, 8.44)	6.72 (5.83, 8.12)	0.5942	1	0.6261	1
S100A8 NK	1.31 (1.13, 1.59)	1.21 (0.79, 1.74)	2.18 (1.23, 2.44)	1.31 (0.88, 1.75)	0.5520	1	0.3299	1
STMN1 NK	2.34 (1.64, 2.34)	1.3 (1.08, 1.55)	2.15 (1.41, 2.16)	1.49 (1.06, 2.03)	0.3953	1	0.4168	1
**monocyte subtype**								
classical monocyte	53.75 (43.01, 59.05)	51.32 (49.34, 54.36)	60.12 (58.45, 61.06)	53.19 (49.24, 55.81)	0.1061	1	0.0513	0.7698
intermediate monocyte	24.31 (23.78, 24.34)	20.2 (19.09, 23.86)	23.99 (23.1, 25)	23.26 (20.2, 24.34)	0.4856	1	0.6261	1
non classical monocyte	21.88 (16.61, 33.18)	25.42 (22.2, 28.09)	14.95 (14.88, 15.79)	23.14 (20.61, 28.48)	0.1632	1	0.1044	1
**B cell subtype**								
naïve B	81.51 (76.37, 81.97)	78.31 (77.76, 79.09)	81.11 (78.11, 82.87)	78.82 (77.71, 80.7)	0.3495	1	0.2556	1
memory B	15.91 (15.39, 20.16)	19.34 (18.38, 19.88)	15.03 (12.5, 15.45)	19.11 (16.06, 20.19)	0.0599	0.8982	0.0348	0.5213
plasmablast	2.7 (2.64, 3.53)	2.43 (1.9, 3.08)	5.72 (2.12, 6.71)	2.7 (2.33, 3.35)	0.3495	1	0.2556	1
IGHV1-18 B	3.69 (3.16, 3.96)	2.11 (1.59, 3.03)	3.2 (2.59, 3.75)	2.64 (2.11, 3.96)	0.4589	1	0.7453	1
IGHV1-2 B	4.51 (3.66, 5.02)	5.17 (4.35, 5.52)	5.24 (5.06, 5.6)	4.9 (3.66, 5.5)	0.4236	1	0.2556	1
IGHV3- 21 B	5.94 (5.55, 6.01)	5.58 (4.28, 6.47)	6.39 (6.37, 7.33)	5.94 (5.13, 6.06)	0.3029	1	0.1439	1
IGHV3-23 B	5.54 (4.41, 5.84)	4.25 (3.38, 5.03)	5.52 (3.88, 6.37)	4.9 (3.44, 5.5)	0.3953	1	0.4168	1
IGHV3-30 B	6.57 (6.26, 11.86)	7.37 (5.59, 9.9)	9.48 (9.13, 10.97)	6.57 (5.8, 10.7)	0.4037	1	0.2556	1
IGHV30-43D B	4.75 (4.53, 5.19)	4.65 (3.23, 6.2)	3.2 (2.59, 6.33)	4.75 (3.95, 5.67)	0.9171	1	0.7453	1
IGHV3-53 B	2.25 (1.6, 2.31)	3.62 (3, 3.78)	1.37 (0.84, 1.93)	2.37 (1.73, 3.62)	0.2893	1	0.2556	1
IGHV5-51 B	2.82 (2.33, 2.95)	2.22 (0.9, 3.3)	3.04 (2.62, 3.45)	2.82 (1.52, 3.15)	0.6707	1	0.4168	1
IGKV3-11 B	6.33 (6.04, 7.16)	7.4 (6.76, 7.88)	4.57 (3.8, 8.29)	6.99 (6.2, 7.89)	0.7778	1	0.6261	1
IGKV3-15 B	14.55 (11.5, 15.73)	15.12 (13, 17.79)	12.36 (11.64, 12.66)	14.55 (12.86, 17.01)	0.2624	1	0.1939	1
IGKV3-20 B	10.33 (9.88, 10.71)	10.51 (9.71, 11.26)	11.05 (10.5, 11.39)	10.33 (9.67, 11.1)	0.7778	1	0.6261	1
IGLC3 B	21.13 (20.19, 21.42)	22.04 (19.58, 25.79)	20.97 (19.89, 21.12)	21.13 (19.56, 23.1)	0.9331	1	1	1
IGLV2-14 B	9.43 (8.28, 10.11)	6.31 (5.95, 6.9)	7.59 (6.85, 7.76)	7.12 (6.31, 9.06)	0.1660	1	0.8708	1
IGLV3-1 B	1.85 (1.63, 2.56)	2.26 (1.75, 2.72)	2.53 (2.28, 2.62)	2.1 (1.63, 2.85)	0.6517	1	0.4168	1
**DC subtype**								
DC1	5.68 (4.36, 6.22)	5.52 (3.22, 6.77)	6.56 (2.56, 7.35)	5.68 (3.66, 6.75)	0.9142	1	0.7453	1
DC2	51.52 (47.56, 56.44)	48.62 (45.82, 52.27)	47.54 (43.64, 51.28)	50 (45.42, 55.3)	0.5942	1	0.6261	1
DC3	19.55 (19.43, 20.38)	19.13 (18.46, 19.65)	19.44 (16.39, 23.08)	19.55 (18.97, 19.66)	0.8865	1	1	1
DC4	5.68 (5.1, 5.87)	6.77 (4.14, 9.5)	4.92 (4.41, 5.13)	5.68 (4.53, 7.52)	0.6601	1	0.4168	1
DC5	3.01 (1.5, 4.53)	0.61 (0, 2.15)	1.47 (1.39, 2.56)	1.23 (0, 3.95)	0.6252	1	0.9341	1
DC6	12.12 (10.04, 17.34)	16.67 (16.64, 18.68)	19.44 (19.12, 24.59)	16.67 (14.34, 19.61)	0.3141	1	0.2548	1

Table S7. Proportion of cell types in the three groups A, B, and C of *M. intracellulare* patients

Cell type	A (n=2)	B (n=2)	C (n=4)	A+B (n=4)	A vs B vs C		A+B vs C	
					p-value	*q*-value	*p*-value	*q*-value
**Total cell**								
T	63.97 (61.6, 66.35)	67.47 (63.09, 71.85)	61.66 (57.71, 67.24)	63.97 (59.09, 70.6)	0.6731	1	0.4705	1
NK	24.95 (24.77, 25.12)	19.89 (18.43, 21.35)	13.29 (12.8, 15.31)	23.7 (21.35, 24.77)	0.0771	0.3856	0.0606	0.3030
Monocyte	7.08 (5.35, 8.81)	7.68 (5.44, 9.93)	16.28 (10.78, 19.58)	7.08 (3.52, 10.94)	0.2231	1	0.1124	0.5618
B	3.4 (2.57, 4.23)	3.82 (3.16, 4.49)	8.34 (5.99, 9.19)	3.77 (2.31, 5.08)	0.4724	1	0.3123	1
DC	0.6 (0.6, 0.6)	1.13 (1.12, 1.14)	0.94 (0.63, 1.26)	0.86 (0.6, 1.12)	0.3998	1	0.6650	1
**T cell subtype**								
gamma delta T	7.58 (7.15, 8)	6.45 (5.95, 6.95)	10.59 (9.69, 11.29)	7.09 (6.41, 7.7)	0.1076	1	0.0606	1
naïve CD8	8.11 (7.63, 8.58)	3.48 (3.01, 3.95)	4.85 (4.11, 5.55)	5.79 (3.95, 7.63)	0.1774	1	0.8852	1
cytotoxic CD8	21.29 (18.68, 23.9)	15.99 (14.57, 17.41)	38.64 (30.26, 48.58)	17.45 (15.35, 20.74)	0.0639	1	0.0304	1
MAIT	5.05 (4.05, 6.06)	3.64 (3.47, 3.82)	3.91 (3.54, 4.14)	3.64 (3.24, 4.76)	0.9200	1	1	1
naïve CD4 T	36.65 (36.47, 36.83)	49.81 (49.27, 50.35)	25.2 (20.46, 29.24)	42.88 (36.83, 49.27)	0.0498	1	0.0304	1
central memory CD4 T	14.11 (13.66, 14.56)	15.15 (14.55, 15.75)	12.99 (10.14, 15.34)	14.48 (13.76, 15.35)	0.6065	1	0.6650	1
Treg	7.21 (6.29, 8.13)	5.48 (5.34, 5.61)	4.02 (3.74, 4.1)	5.56 (5.32, 6.57)	0.0639	1	0.0304	1
GZMH cytotoxic CD8	65.94 (64.33, 67.54)	71.12 (70.79, 71.45)	62.14 (61.27, 66.3)	69.8 (67.54, 70.79)	0.4083	1	0.4705	1
GZMK cytotoxic CD8	34.06 (32.46, 35.67)	28.88 (28.55, 29.21)	37.86 (33.7, 38.73)	30.2 (29.21, 32.46)	0.4083	1	0.4705	1
FOXP3 Treg	41.63 (41.49, 41.77)	39.14 (30.03, 48.26)	70.6 (67.19, 71.73)	41.63 (36.24, 45.78)	0.0695	1	0.0304	1
MALAT1 Treg	58.37 (58.23, 58.51)	60.86 (51.74, 69.97)	29.4 (28.27, 32.81)	58.37 (54.22, 63.76)	0.0695	1	0.0304	1
**NK cell subtype**								
adaptive memory like NK	43.4 (41.71, 45.1)	40.34 (37.6, 43.09)	28.14 (26.1, 35.11)	42.92 (38.72, 46.07)	0.4724	1	0.3123	1
cytotoxic NK	42.79 (42.1, 43.49)	47.26 (45.63, 48.89)	53.93 (47.37, 57.15)	44.09 (43.35, 45.77)	0.4724	1	0.3123	1
NKT	6.5 (5.49, 7.52)	5.18 (4.55, 5.8)	7.7 (6.75, 8.45)	5.45 (4.34, 6.95)	0.4083	1	0.4705	1
regulatory NK	5.38 (5.27, 5.49)	5.48 (4.89, 6.07)	6.86 (6.02, 7.12)	5.38 (4.95, 5.86)	0.5134	1	0.3123	1
S100A8 NK	0.81 (0.81, 0.81)	0.64 (0.56, 0.71)	1.42 (1.04, 1.56)	0.8 (0.71, 0.81)	0.3679	1	0.3123	1
STMN1 NK	1.11 (1.01, 1.21)	1.1 (1.08, 1.13)	1.14 (1.12, 1.4)	1.1 (1.02, 1.19)	0.6731	1	0.4705	1
**monocyte subtype**								
classical monocyte	45.09 (42.18, 48)	45.36 (42.72, 47.99)	53.62 (53.29, 53.99)	45.36 (39.88, 50.7)	0.0695	1	0.0304	0.4557
intermediate monocyte	18.75 (14.28, 23.22)	23.3 (21.96, 24.65)	26.8 (24.82, 29.02)	23.3 (17.91, 26.42)	0.6731	1	0.4705	1
non classical monocyte	36.16 (28.78, 43.54)	31.34 (30.05, 32.63)	19.61 (17.2, 21.72)	31.34 (26.92, 38.17)	0.1271	1	0.0606	0.9090
**B cell subtype**								
naïve B	77.32 (73.77, 80.87)	74.94 (71.08, 78.8)	70.45 (63.32, 77.57)	76.44 (69.47, 83.1)	0.4724	1	0.3123	1
memory B	17.75 (15.99, 19.52)	19.59 (17.16, 22.02)	19.96 (16.72, 24.19)	18 (14.6, 22.07)	0.5698	1	0.4705	1
plasmablast	4.93 (3.14, 6.72)	5.47 (4.04, 6.9)	8.93 (5.71, 11.83)	5.47 (2.3, 8.38)	0.5134	1	0.3123	1
IGHV1-18 B	1.71 (0.85, 2.56)	2.5 (1.68, 3.31)	1.91 (1.07, 2.43)	2.14 (0.65, 3.6)	0.7972	1	0.7715	1
IGHV1-2 B	4.28 (3.39, 5.17)	7.02 (6.82, 7.23)	3.4 (2.43, 4.01)	6.33 (5.17, 6.82)	0.1271	1	0.1939	1
IGHV3- 21 B	6.11 (6.08, 6.13)	8.26 (7.44, 9.09)	5.65 (5.21, 6.34)	6.38 (6.13, 7.44)	0.2096	1	0.1939	1
IGHV3-23 B	6.67 (6.37, 6.98)	6.2 (5.99, 6.4)	6.22 (5.32, 6.7)	6.33 (5.99, 6.78)	0.6065	1	0.6650	1
IGHV3-30 B	7.47 (6.77, 8.18)	6.21 (5.59, 6.84)	9.66 (7.02, 10.71)	6.77 (5.79, 7.82)	0.4724	1	0.3123	1
IGHV30-43D B	3 (2.98, 3.01)	4.8 (4.46, 5.13)	4.47 (2.43, 5.81)	3.58 (3.01, 4.46)	0.3998	1	0.6650	1
IGHV3-53 B	2.2 (1.78, 2.61)	1.81 (1.48, 2.15)	2.28 (1.89, 2.8)	1.92 (1.31, 2.62)	0.9200	1	1	1
IGHV5-51 B	0.46 (0.23, 0.68)	2.95 (2.77, 3.13)	3.22 (2.8, 3.7)	1.75 (0.68, 2.77)	0.1054	1	0.1124	1
IGKV3-11 B	4.59 (3.81, 5.37)	5.77 (5.76, 5.78)	5.12 (4.49, 6.27)	5.77 (5.07, 5.88)	0.7952	1	0.8852	1
IGKV3-15 B	20.66 (18.87, 22.45)	13.44 (11.89, 14.98)	13.42 (13.15, 14.25)	16.81 (14.98, 18.87)	0.1353	1	0.3123	1
IGKV3-20 B	8.01 (5.52, 10.5)	11.03 (9.65, 12.41)	9.71 (8.94, 10.59)	10.62 (6.96, 13.19)	0.7952	1	0.8852	1
IGLC3 B	26.12 (22.51, 29.73)	20.53 (20.47, 20.6)	21.28 (20.05, 24.28)	20.53 (20.03, 23.83)	0.7952	1	0.8852	1
IGLV2-14 B	7.47 (6.77, 8.18)	8.33 (7.47, 9.2)	9.3 (8.61, 11.47)	7.75 (6.47, 9.18)	0.5698	1	0.4705	1
IGLV3-1 B	1.25 (0.63, 1.88)	1.15 (0.57, 1.72)	2.68 (2.15, 3.4)	1.15 (0, 2.35)	0.4987	1	0.3094	1
**DC subtype**								
DC1	3.57 (1.79, 5.36)	5.72 (4.64, 6.79)	4.42 (2.88, 5.45)	5.36 (2.68, 7.32)	0.7972	1	0.7715	1
DC2	60.71 (51.79, 69.64)	42.81 (42.19, 43.43)	33.59 (29.89, 40.82)	43.45 (42.54, 52.68)	0.2925	1	0.1939	1
DC3	20.54 (17.41, 23.66)	15.04 (14.82, 15.26)	18.86 (14.7, 22.81)	15.04 (14.53, 18.3)	0.9200	1	1	1
DC4	2.68 (1.34, 4.02)	8.59 (7.27, 9.92)	7.16 (5.98, 8.19)	5.65 (4.02, 7.27)	0.2231	1	0.6650	1
DC5	0.89 (0.45, 1.34)	2.88 (2.63, 3.12)	2.53 (1.88, 2.79)	2.08 (1.34, 2.63)	0.3153	1	0.5614	1
DC6	11.61 (9.38, 13.84)	24.96 (23.15, 26.77)	30.84 (26.46, 35.57)	18.71 (13.84, 23.15)	0.1054	1	0.1124	1

Table S8. Proportion of cell types in 16 MAC-PD patients excluding male and non-NB patients

Cell type	A (n=4)	B (n=5)	C (n=7)	A+B (n=9)	C-1 (n=2)	C-2 (n=2)	A vs B vs C		A+B vs C		C-1 vs C-2	
							p-value	*q*-value	*p*-value	*q*-value	*p*-value	*q*-value
**Total cell**												
T	66.42 (63.77, 69.28)	68.27 (55.37, 73.78)	64.88 (59.56, 66.45)	67.56 (59.22, 73.78)	68.07 (66.82, 69.31)	62.78 (61.73, 63.83)	0.7099	1	0.4587	1	0.2453	1
NK	16.46 (13.44, 20.27)	16.97 (14.86, 23.89)	12.45 (10.48, 13.29)	16.97 (14.08, 23.89)	7.7 (7.03, 8.37)	13.78 (12.85, 14.71)	0.1156	0.5779	0.0443	0.2215	0.2453	1
Monocyte	9.6 (8.04, 11.43)	14.55 (7.46, 15.47)	15.66 (12.62, 19.07)	10.53 (7.46, 14.55)	17.18 (16.42, 17.93)	12.44 (12.11, 12.78)	0.1055	0.5277	0.0567	0.2837	0.2453	1
B	5.54 (4.77, 6.19)	3.88 (2.8, 4.52)	7.99 (7.37, 9.48)	4.52 (3.88, 6.02)	6 (5.01, 6.99)	9.97 (9.43, 10.5)	0.0140	0.0702	0.0059	0.0296	0.2453	1
DC	0.7 (0.58, 0.94)	1.13 (1.12, 1.39)	1.15 (0.8, 1.29)	1.12 (0.8, 1.37)	1.06 (0.9, 1.22)	1.02 (0.94, 1.11)	0.1856	0.9281	0.8323	1	1	1
**T cell subtype**												
gamma delta T	9.11 (8.14, 10.98)	6.72 (5.45, 9.83)	10.4 (7.67, 11.5)	8.43 (6.72, 9.83)	6.37 (6.34, 6.41)	11.31 (10.85, 11.76)	0.6289	1	0.5966	1	0.2453	1
naïve CD8	4.77 (3.04, 6.62)	5.27 (4.43, 5.72)	7.19 (5.26, 7.84)	5.27 (3.09, 6.45)	7.84 (7.82, 7.86)	6.65 (5.83, 7.47)	0.5298	1	0.2898	1	1	1
cytotoxic CD8	23.51 (19.92, 27.9)	26.88 (13.15, 39.31)	24.88 (22.11, 29.8)	26.5 (18.14, 32.11)	16.79 (12.75, 20.83)	26.69 (24.67, 28.7)	0.9355	1	0.9157	1	0.6985	1
MAIT	3.43 (3.1, 4.03)	3.12 (2.95, 3.99)	3.79 (2.7, 5.22)	3.12 (3.05, 3.99)	4.53 (3.58, 5.48)	3.05 (2.68, 3.42)	0.9678	1	1	1	0.6985	1
naïve CD4 T	38.64 (34.87, 41.11)	34.68 (27.32, 45.12)	33.73 (29.66, 38.84)	36.28 (30.61, 41.43)	42.92 (41.16, 44.69)	34.39 (32.44, 36.34)	0.7446	1	0.5966	1	0.2453	1
central memory CD4 T	13.19 (12.87, 14.69)	16.35 (11.04, 17.31)	15.29 (13.03, 15.94)	13.21 (12.01, 17.31)	15.98 (15.33, 16.62)	13.33 (12.35, 14.31)	0.9606	1	0.9157	1	0.6985	1
Treg	5.52 (5.07, 5.83)	4.57 (3.92, 4.73)	4.3 (4.16, 5.32)	4.73 (4.19, 5.68)	5.57 (4.93, 6.2)	4.59 (4.44, 4.73)	0.4293	1	1	1	0.6985	1
GZMH cytotoxic CD8	66.97 (64.13, 68.31)	61.63 (60.38, 66.11)	60.71 (57.04, 62.14)	65.92 (60.38, 68.03)	45.16 (38.73, 51.6)	65.53 (63.12, 67.93)	0.4102	1	0.2443	1	0.2453	1
GZMK cytotoxic CD8	33.03 (31.69, 35.87)	38.37 (33.89, 39.62)	39.29 (37.86, 42.96)	34.08 (31.97, 39.62)	54.84 (48.4, 61.27)	34.47 (32.07, 36.88)	0.4102	1	0.2443	1	0.2453	1
FOXP3 Treg	41.78 (38.27, 48.69)	56.21 (50, 58.93)	64.49 (57.43, 70.6)	50 (41.35, 58.93)	69.99 (67.24, 72.74)	56.89 (50.49, 63.28)	0.1172	1	0.0567	1	0.6985	1
MALAT1 Treg	58.22 (51.31, 61.73)	43.79 (41.07, 50)	35.51 (29.4, 42.57)	50 (41.07, 58.65)	30.01 (27.26, 32.76)	43.11 (36.72, 49.51)	0.1172	1	0.0567	1	0.6985	1
**NK cell subtype**												
adaptive memory like NK	34.49 (28.48, 40.75)	30.92 (28.67, 34.85)	28.32 (26.16, 32.45)	30.92 (28.67, 40.01)	30.47 (28.18, 32.77)	29.08 (28.7, 29.46)	0.5386	1	0.2898	1	1	1
cytotoxic NK	49.43 (43.36, 55.72)	55.85 (50.53, 56.29)	55.17 (43.96, 59.46)	54.67 (44.19, 56.29)	47.65 (41.96, 53.33)	53.4 (52.52, 54.28)	0.8195	1	0.9157	1	1	1
NKT	5.33 (4.2, 6.94)	5.8 (5.76, 6.42)	7.81 (4.61, 11.95)	5.8 (4.25, 6.42)	8.17 (5.08, 11.25)	7.66 (6.31, 9.01)	0.5995	1	0.3408	1	1	1
regulatory NK	6.78 (5.39, 8.22)	6.66 (5.6, 6.72)	6.77 (5.77, 7.69)	6.66 (5.46, 8.1)	8.21 (7.46, 8.96)	5.89 (5.36, 6.42)	0.8236	1	0.7508	1	0.6985	1
S100A8 NK	1.13 (0.92, 1.45)	0.81 (0.75, 1.62)	1.5 (0.87, 2.09)	0.95 (0.81, 1.62)	1.84 (1.53, 2.14)	2.35 (1.24, 3.46)	0.7955	1	0.5966	1	1	1
STMN1 NK	1.82 (1.21, 2.34)	1.29 (1.11, 1.49)	1.41 (1.14, 2.15)	1.3 (1.11, 1.73)	3.67 (2.91, 4.43)	1.62 (1.35, 1.88)	0.6245	1	0.6720	1	0.2453	1
**monocyte subtype**												
classical monocyte	52.33 (46.25, 56.4)	49.45 (49.13, 53.19)	55.9 (54.03, 60.59)	50.91 (49.13, 53.75)	62.97 (62.02, 63.93)	57.03 (55.49, 58.57)	0.0803	1	0.0343	0.5139	0.2453	1
intermediate monocyte	24.34 (24.05, 25.2)	21.28 (19.12, 25.15)	23.99 (22.08, 25.76)	24.31 (21.28, 25.15)	23.54 (23.32, 23.77)	26.05 (25.52, 26.57)	0.4685	1	0.9157	1	0.2453	1
non classical monocyte	21.64 (18.89, 27.53)	27.69 (25.73, 29.27)	18.97 (14.92, 21.64)	25.73 (21.88, 29.27)	13.48 (12.75, 14.22)	16.92 (15.9, 17.94)	0.0521	0.7815	0.0567	0.8511	0.6985	1
**B cell subtype**												
naïve B	81.97 (78.94, 82.93)	77.97 (77.8, 78.82)	78.11 (77.32, 81.38)	78.82 (77.8, 81.51)	80.49 (79.3, 81.68)	82.63 (81.26, 84)	0.4648	1	1	1	0.6985	1
memory B	15.39 (14.71, 18.04)	20.34 (19.11, 20.79)	16.16 (15.24, 16.61)	19.11 (15.91, 20.79)	15.6 (15.32, 15.88)	14.44 (13.47, 15.41)	0.1612	1	0.4587	1	1	1
plasmablast	2.64 (2.27, 3.11)	2.07 (1.69, 3.91)	5.72 (2.93, 6.54)	2.58 (1.69, 3.91)	3.91 (3, 4.82)	2.93 (2.53, 3.33)	0.2603	1	0.1123	1	1	1
IGHV1-18 B	3.55 (3.22, 3.82)	2.4 (1.82, 4.13)	2.52 (2.26, 3.17)	3.42 (2.4, 4.13)	2.35 (2.23, 2.47)	3.61 (3.06, 4.15)	0.4790	1	0.5966	1	0.6985	1
IGHV1-2 B	3.66 (2.74, 4.77)	5.45 (4.9, 5.71)	5.06 (4.27, 5.3)	4.9 (2.82, 5.54)	5.33 (5.2, 5.47)	4.1 (3.67, 4.54)	0.4159	1	0.9157	1	0.2453	1
IGHV3- 21 B	6.01 (5.75, 6.09)	6.96 (6.06, 7.69)	6.37 (5.75, 7.46)	6.07 (5.94, 6.96)	7.46 (7.39, 7.53)	7.04 (6.42, 7.66)	0.5206	1	0.8323	1	1	1
IGHV3-23 B	5.84 (4.98, 6.43)	5.45 (4.9, 5.79)	6.37 (5.64, 6.72)	5.54 (4.9, 6.15)	5.53 (4.7, 6.35)	5.64 (5.58, 5.7)	0.4344	1	0.2898	1	1	1
IGHV3-30 B	7.73 (6.42, 10.95)	8.26 (5.66, 9.09)	9.96 (9.42, 11.96)	8.26 (5.94, 9.09)	10.23 (9.85, 10.6)	13.24 (13.1, 13.39)	0.1326	1	0.0567	1	0.2453	1
IGHV30-43D B	4.53 (3.97, 4.97)	4.13 (2.1, 5.71)	5.69 (2.91, 6.24)	4.3 (2.96, 5.63)	4.46 (3.52, 5.39)	2.72 (2.47, 2.98)	0.7831	1	0.5254	1	0.6985	1
IGHV3-53 B	1.81 (1.26, 2.28)	3.48 (2.48, 3.64)	1.93 (0.96, 2.28)	2.37 (1.37, 3.48)	0.64 (0.53, 0.74)	2.05 (1.99, 2.1)	0.1400	1	0.2443	1	0.2453	1
IGHV5-51 B	2.33 (1.61, 2.88)	3.23 (1.74, 3.31)	2.85 (2.65, 3.24)	2.82 (1.74, 3.23)	2.78 (2.44, 3.11)	3.32 (3.18, 3.46)	0.4694	1	0.4587	1	0.6985	1
IGKV3-11 B	6.24 (6.05, 6.74)	6.52 (6.06, 6.99)	4.55 (4.06, 6.99)	6.33 (6.06, 6.99)	6.42 (5.11, 7.74)	6.3 (5.31, 7.29)	0.3567	1	0.1688	1	1	1
IGKV3-15 B	15.73 (13.02, 16.95)	16.53 (13.13, 17.12)	12.66 (12, 13.13)	16.53 (13.13, 17.08)	12.15 (11.89, 12.4)	11.81 (10.88, 12.74)	0.1326	1	0.0567	1	1	1
IGKV3-20 B	10.71 (10.1, 11.56)	9.91 (9.09, 11.11)	11.05 (9.71, 11.63)	10.33 (9.43, 11.11)	12.16 (11.78, 12.55)	11.46 (11.25, 11.67)	0.6665	1	0.8323	1	0.6985	1

IGLC3 B	20.19 (19.17, 21.28)	19.6 (19.52, 20.66)	20.86 (19.86, 21.05)	19.6 (19.26, 21.13)	20.48 (20.15, 20.8)	18.76 (18.19, 19.32)	0.8751	1	0.7508	1	0.6985	1
IGLV2-14 B	9.16 (8.44, 9.77)	6.61 (6.31, 8.69)	7.87 (7.68, 8.59)	8.69 (6.61, 9.43)	7.68 (7.64, 7.72)	7.36 (6.72, 7.99)	0.3852	1	0.7508	1	1	1
IGLV3-1 B	2.18 (1.74, 2.7)	1.3 (0.7, 2.1)	2.53 (2.16, 2.83)	1.85 (1.3, 2.42)	2.34 (2.25, 2.44)	2.6 (2.38, 2.82)	0.0521	1	0.0567	1	0.6985	1
**DC subtype**												
DC1	6.22 (5.02, 6.86)	3.57 (2.29, 4.29)	6.56 (4.42, 7.09)	4.29 (3.03, 6.77)	8.22 (7.79, 8.66)	3.21 (2.88, 3.53)	0.2558	1	0.2898	1	0.2453	1
DC2	47.56 (43.42, 53.98)	50 (47.24, 59.09)	42.65 (33.59, 45.59)	50 (44.05, 59.09)	43.14 (42.89, 43.39)	44.23 (40.71, 47.76)	0.0803	1	0.0343	1	1	1
DC3	20.38 (19.49, 22.61)	19.63 (18.63, 19.7)	22.73 (15.7, 23.08)	19.63 (19.32, 21.21)	19.77 (17.16, 22.39)	23.08 (23.08, 23.08)	0.7176	1	0.9156	1	1	1
DC4	5.52 (5.15, 5.78)	4.58 (4.55, 5.95)	5.13 (4.66, 7.16)	5.36 (4.55, 5.95)	2.21 (1.1, 3.31)	7.69 (6.41, 8.97)	0.9606	1	0.9157	1	0.2453	1
DC5	2.4 (1.34, 3.77)	1.23 (0, 2.38)	2.5 (0.74, 2.56)	1.79 (0, 3.01)	2.55 (2.01, 3.09)	2.56 (2.56, 2.56)	0.6717	1	1	1	1	1
DC6	14.1 (11.08, 17.69)	16.67 (16.56, 16.67)	24.59 (21.1, 31.59)	16.56 (12.12, 16.67)	24.1 (21.61, 26.6)	19.23 (17.31, 21.15)	0.0614	1	0.0261	0.7834	0.6985	1

Supplementary Figures

Supplementary Figures legends

Fig. S1 Clustering of 189,779 single cells from MAC-PD patients

(a) UMAP plot colored by cluster. **(b)** Heat map of the gene expression pattern for the top 10 genes per cell type. **(c)** Dot plot of highly featured genes expressed in each cell types. The size of the dot corresponds to the percentage of cells expressing the gene in each cell type. The color presents the average expression level. **(d)** Expression of known marker genes for the cell types on the UMAP plot. **(e)** UMAP plots of single-cell profiles with each cell color-coded by cell type (left to right: split by group). **(f)** Cell number and proportion of five main cells from PBMCs of each sample.

Fig. S2 DEGs analysis of five main cell types in each group comparison

(a) Scatter plot showing DEGs between group A and B. Each dot indicates an individual gene, colored by red when a gene is a significant DEG. **(b)** Scatter plot showing DEGs between group A and C. **(c)** Scatter plot showing DEGs between group B and C. **(d)** Scatter plot showing DEGs between no-treatment group (groups A and B) and treatment group C. **(e)** Violin plot showing expression levels of DEGs between no-treatment group (groups A and B) and treatment group C.

Fig. S3 T cell subsets analysis

(a) UMAP plot colored by cluster. **(b)** Heat map of the gene expression pattern for the top 10 genes per T cell subset. **(c)** Dot plot indicating expression of key genes for each T cell subset. **(d)** UMAP Plot colored by T cell subsets, split by group. **(e)** Cell number and relative

proportion of T cell subsets from PBMCs of each sample. **(f)** GO analysis of DEGs in MALAT^{+} Tregs. Dot plot showing the most significant GO terms. The top 20 GO terms are ordered on the y-axis. The x-axis represents the gene percentage in enriched GO terms. The sizes of the dots represent the gene ratio included in each GO term. The color gradient of dots represents the adjusted p-values of each enriched GO term.

Fig. S4 TCR clonotype analysis

(a) The productivity of TCR α and β chain pairs in all T cells of each sample. **(b)** UMAP plot showing all T cells from the 10× Genomics dataset after re-clustering, with cells with TCR information shown in color. Each color represents a distinct TCR clonotype. **(c)** Distribution of the TCR clonotype frequency by each patient. **(d)** Distribution of the TCR clonotype frequency by each group. **(e)** Pie charts showing the distribution of TCRαβ clonotypes of all T cells in each sample based on clonal frequency. The paired CDR3α and CDR3β sequences with clonal frequency >50 (the dominant clonotype) in each sample are listed below the pie charts.

Fig. S5 NK cell subsets analysis

(a) UMAP plot colored by cluster. **(b)** Heat map of the gene expression pattern for the top 10 genes per NK cell subset. **(c)** Dot plot indicating expression of key genes for each NK cell subset. **(d)** UMAP plot of NK cells, color-coded by their gene expressions (gray to violet). **(e)** UMAP plot colored by NK cell subsets, split by group. **(f)** Cell number and relative proportion of NK cell subsets of each sample. **(g)** GO analysis of DEGs in NK cell subsets compared between group C and combined groups A and B. Dot plot showing the most significant GO terms in each cell type. The top 5 GO terms were selected from each cluster. The sizes of the dots represent the gene ratio included in each GO term. The color gradient of dots represents the adjusted p-values of each enriched GO term. **(h)** Scatter plot showing DEGs between

subgroup C-1 and C-2. Each dot indicates an individual gene, colored by red when a gene is a significant DEG. **(i)** GO analysis of DEGs in NK cell subsets compared between subgroup C-1 and C-2. Dot plot showing the most significant GO terms in each cell type. The top 5 GO terms were selected from each cluster. The sizes of the dots represent the gene ratio included in each GO term. The color gradient of dots represents the adjusted p-values of each enriched GO term.

Fig. S6 Monocyte subsets analysis

(a) UMAP plot colored by cluster. **(b)** Heat map of the gene expression pattern for the top 10 genes per monocyte subset. **(c)** Dot plot of highly featured genes expressed in each cell types. **(d)** UMAP plot indicating expression of key genes for each monocyte subset. **(e)** UMAP plot colored by monocyte subsets, split by group. **(f)** Cell number and relative proportion of monocyte subsets of each sample. **(g)** GO analysis of DEGs in monocyte subsets compared between subgroup C-1 and C-2. Dot plot showing the most significant GO terms in each cell type. The top 10 GO terms were selected from each cluster. The sizes of the dots represent the gene ratio included in each GO term. The color gradient of dots represents the adjusted p-values of each enriched GO term.

Fig. S7 B cell subsets analysis

(a) UMAP plot colored by cluster. **(b)** Heat map of the gene expression pattern for the top 10 genes per B cell subset. **(c)** Dot plot indicating expression of key genes for each B cell subset. **(d)** UMAP plot color-coded by Ig gene expression as indicated. The dominant variable region for B cells was determined by evaluating genes with the highest expression levels. **(e)** UMAP plot colored by B cell subsets, split by group. **(f)** Cell number and relative proportion of B cell subsets from PBMCs of each sample. **(g)** Violin plot showing CD83 expression level between

no-treatment group (groups A and B) and treatment group C. F, Scatter plot showing DEGs between subgroup C-1 and C-2.

Fig. S8 DC subsets analysis

(a) UMAP plot colored by cluster. **(b)** Heat map of the gene expression pattern for the top 10 genes per DC subset. **(c)** Dot plot indicating expression of key genes for each DC subset. **(d)** UMAP plot colored by DC subsets, split by group. **(e)** Cell number and relative proportion of DC subsets from PBMCs of each sample.

Fig. S1

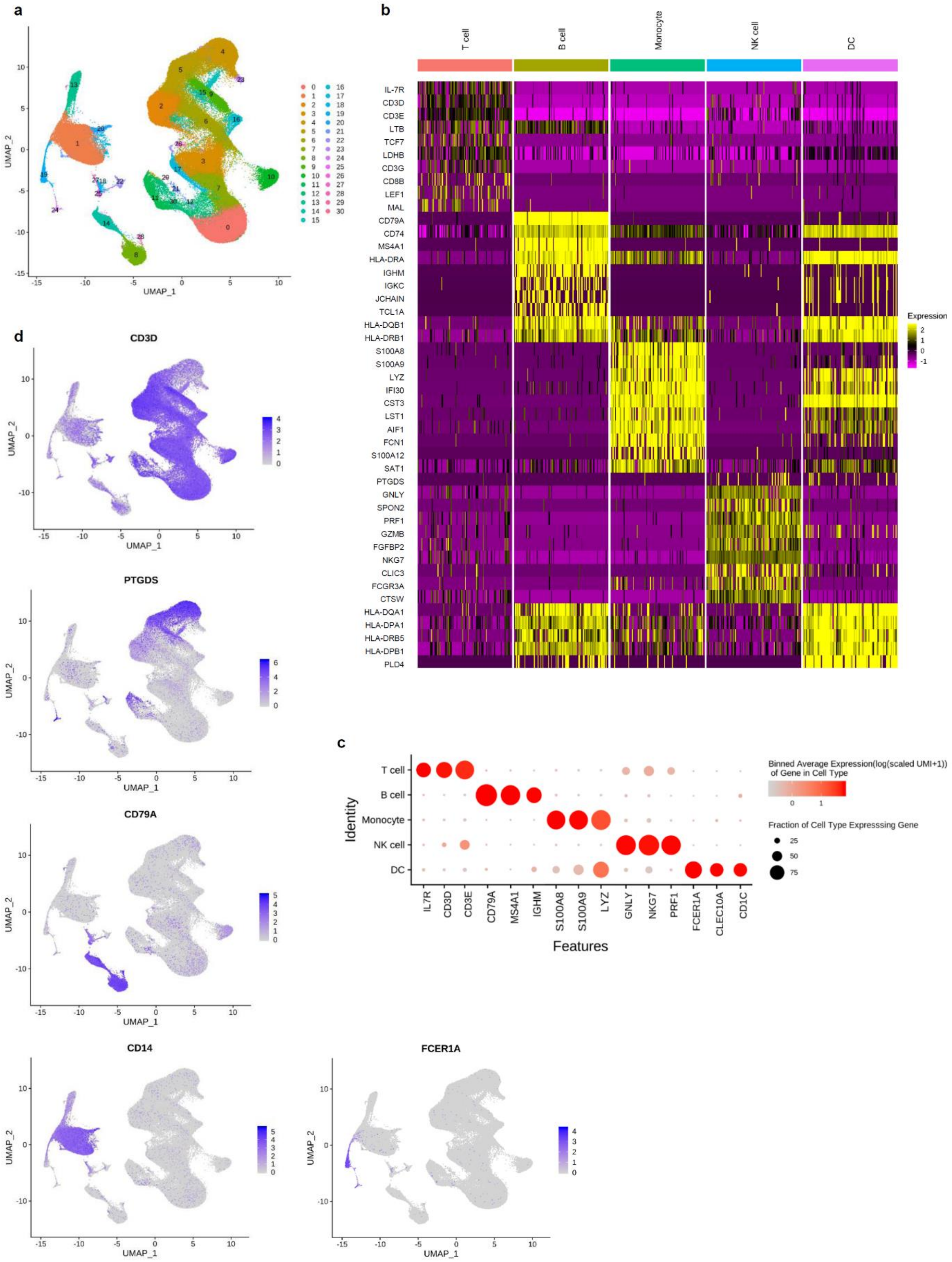
a
UMAP_1
UMAP_2
b
T cell
B cell
Monocyte
NK cell
DC
IL-7R
CD3D
CD3E
LTB
TCF7
LDHB
CD3G
CD8B
LEF1
MAL
CD79A
CD74
MS4A1
HLA-DRA
IGHM
IGKC
JCHAIN
TCL1A
HLA-DQB1
HLA-DRB1
S100A8
S100A9
LYZ
IFI30
CST3
LST1
AIF1
FCN1
S100A12
SAT1
PTGDS
GNLY
SPON2
PRF1
GZMB
FGFBP2
NKG7
CLIC3
FCGR3A
CTSW
HLA-DQA1
HLA-DPA1
HLA-DRB5
HLA-DPB1
PLD4
Expression
c
Identity
Features
IL7R
CD3D
CD3E
CD79A
MS4A1
IGHM
S100A8
S100A9
LYZ
GNLY
NKG7
PRF1
FCER1A
CLEC10A
CD1C
Binned Average Expression(log(scaled UMI+1)) of Gene in Cell Type
Fraction of Cell Type Expresssing Gene
d
CD3D
PTGDS
CD79A
CD14
FCER1A

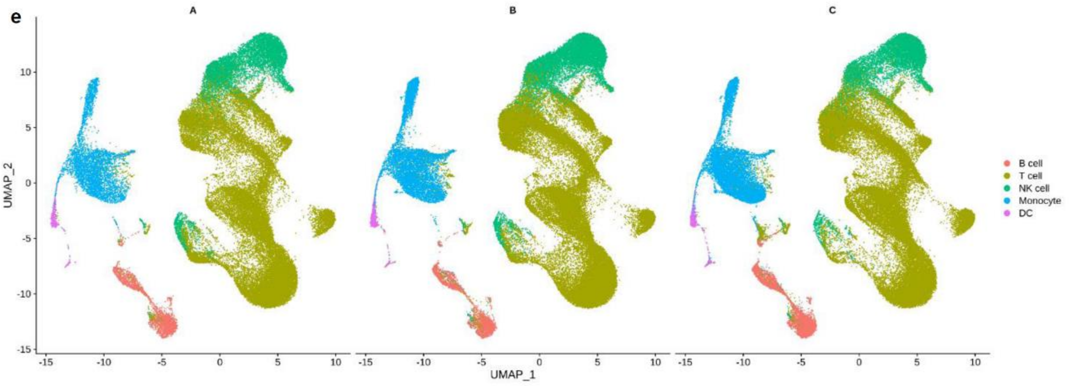
e
A
B
C
UMAP_2
UMAP_1
B cell
T cell
NK cell
Monocyte
DC

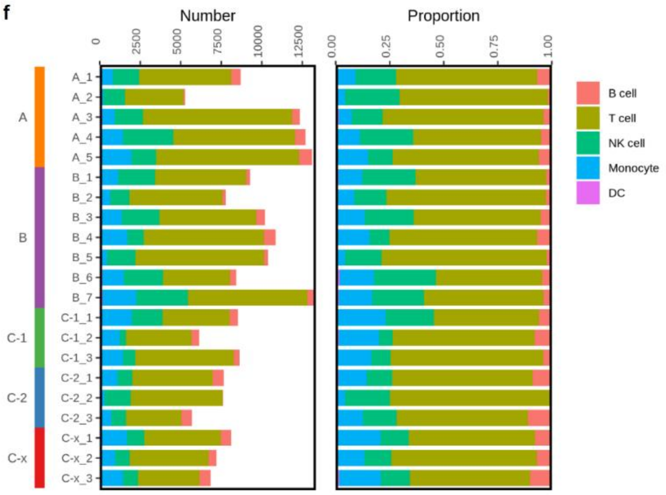
f
Number
Proportion
A
B
C-1
C-2
C-x
B cell
T cell
NK cell
Monocyte
DC

Fig. S2

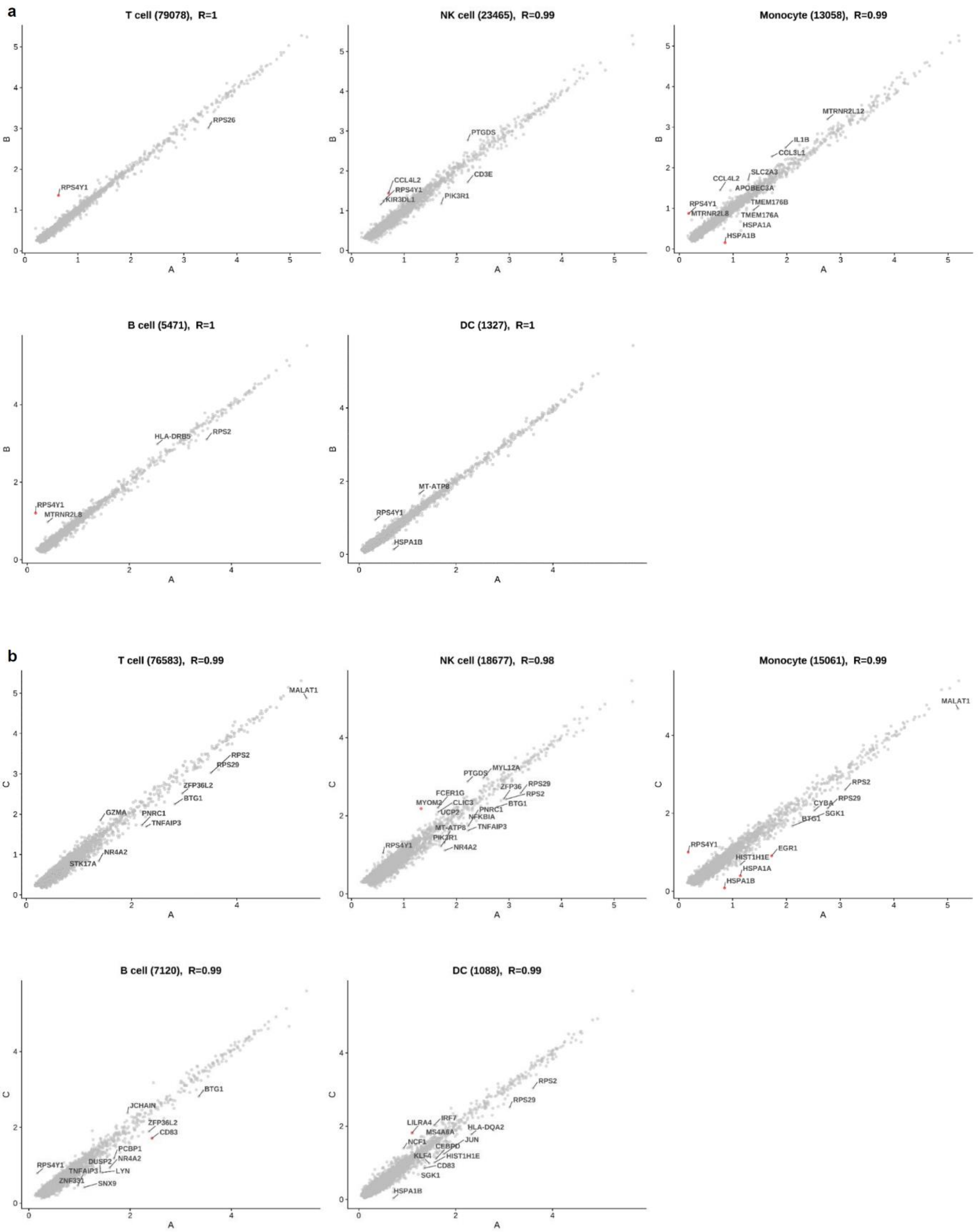
a
T cell (79078), R=1
RPS26
RPS4Y1
NK cell (23465), R=0.99
PTGDS
CD3E
CCL4L2
RPS4Y1
KIR3DL1
PIK3R1
Monocyte (13058), R=0.99
MTRNR2L12
IL1B
CCL3L1
SLC2A3
CCL4L2
APOBEC3A
RPS4Y1
MTRNR2L8
TMEM176B
TMEM176A
HSPA1A
HSPA1B
B cell (5471), R=1
HLA-DRB5
RPS2
RPS4Y1
MTRNR2L8
DC (1327), R=1
MT-ATP8
RPS4Y1
HSPA1B
b
T cell (76583), R=0.99
MALAT1
RPS2
RPS29
ZFP36L2
BTG1
GZMA
PNRC1
TNFAIP3
NR4A2
STK17A
NK cell (18677), R=0.98
PTGDS
MYL12A
FCER1G
MYOM2
CLIC3
UCP2
ZFP36
RPS29
RPS2
BTG1
PNRC1
NFKBIA
MT-ATP8
TNFAIP3
PIK3R1
NR4A2
RPS4Y1
Monocyte (15061), R=0.99
MALAT1
RPS2
CYBA
RPS29
SGK1
BTG1
RPS4Y1
EGR1
HIST1H1E
HSPA1A
HSPA1B
B cell (7120), R=0.99
BTG1
JCHAIN
ZFP36L2
CD83
PCBP1
NR4A2
DUSP2
RPS4Y1
TNFAIP3
LYN
ZNF331
SNX9
DC (1088), R=0.99
RPS2
RPS29
LILRA4
IRF7
MS4A6A
HLA-DQA2
NCF1
JUN
CEBPD
KLF4
HIST1H1E
CD83
SGK1
HSPA1B
A
B
C

c

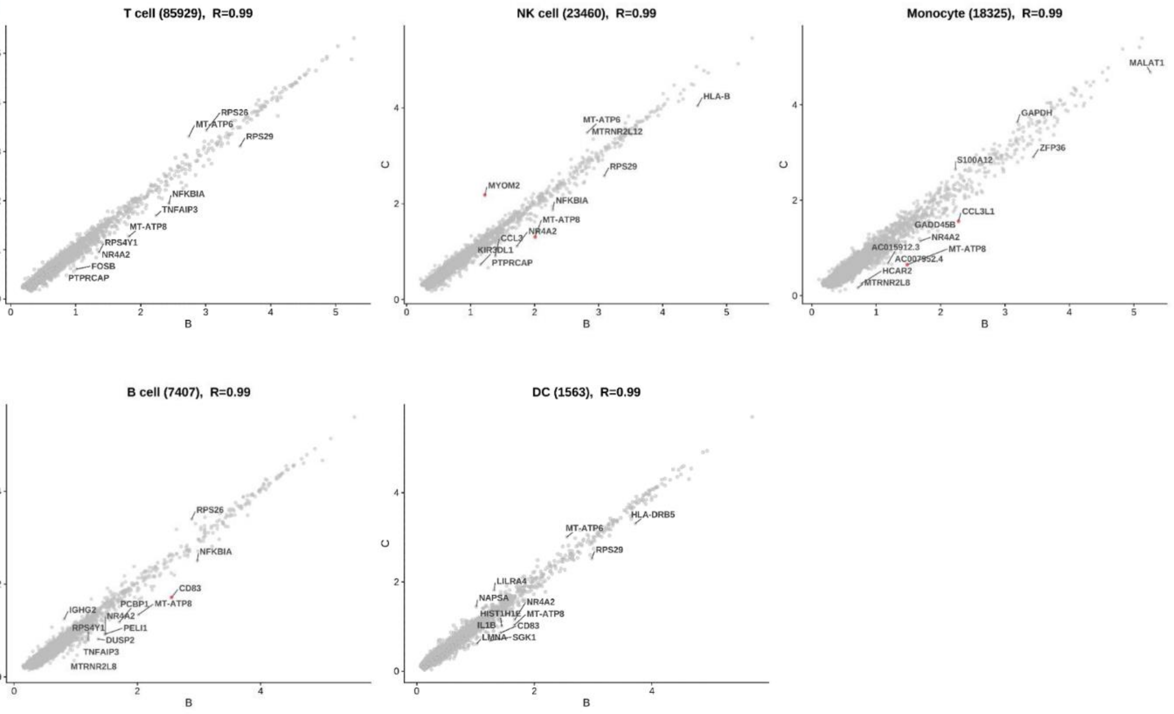
T cell (85929), R=0.99
RPS26
MT-ATP6
RPS29
NFKBIA
TNFAIP3
MT-ATP8
RPS4Y1
NR4A2
FOSB
PTPRCAP
NK cell (23460), R=0.99
HLA-B
MT-ATP6
MTRNR2L12
RPS29
MYOM2
NFKBIA
MT-ATP8
CCL3
NR4A2
KIR3DL1
PTPRCAP
Monocyte (18325), R=0.99
MALAT1
GAPDH
ZFP36
S100A12
CCL3L1
GADD45B
NR4A2
AC015912.3
MT-ATP8
AC007952.4
HCAR2
MTRNR2L8
B cell (7407), R=0.99
RPS26
NFKBIA
CD83
PCBP1
MT-ATP8
IGHG2
NR4A2
RPS4Y1
PELI1
DUSP2
TNFAIP3
MTRNR2L8
DC (1563), R=0.99
HLA-DRB5
MT-ATP6
RPS29
LILRA4
NAPSA
NR4A2
HIST1H1E
MT-ATP8
IL1B
CD83
LMNA
SGK1
C
B

d

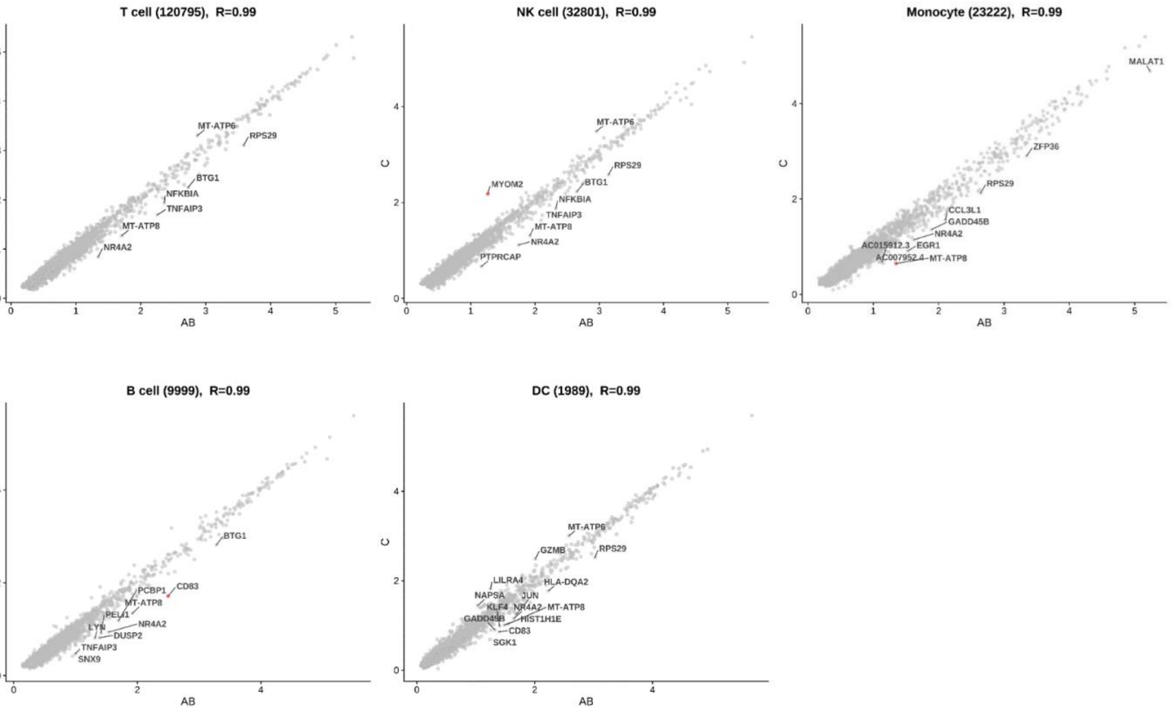
T cell (120795), R=0.99
MT-ATP6
RPS29
BTG1
NFKBIA
TNFAIP3
MT-ATP8
NR4A2
NK cell (32801), R=0.99
MT-ATP6
RPS29
MYOM2
BTG1
NFKBIA
TNFAIP3
MT-ATP8
NR4A2
PTPRCAP
Monocyte (23222), R=0.99
MALAT1
ZFP36
RPS29
CCL3L1
GADD45B
NR4A2
AC015912.3
EGR1
AC007952.4
MT-ATP8
B cell (9999), R=0.99
BTG1
CD83
PCBP1
MT-ATP8
PELI1
LYN
NR4A2
DUSP2
TNFAIP3
SNX9
DC (1989), R=0.99
MT-ATP6
GZMB
RPS29
LILRA4
HLA-DQA2
NAPSA
JUN
KLF4
NR4A2
MT-ATP8
GADD45B
HIST1H1E
CD83
SGK1
C
AB

e

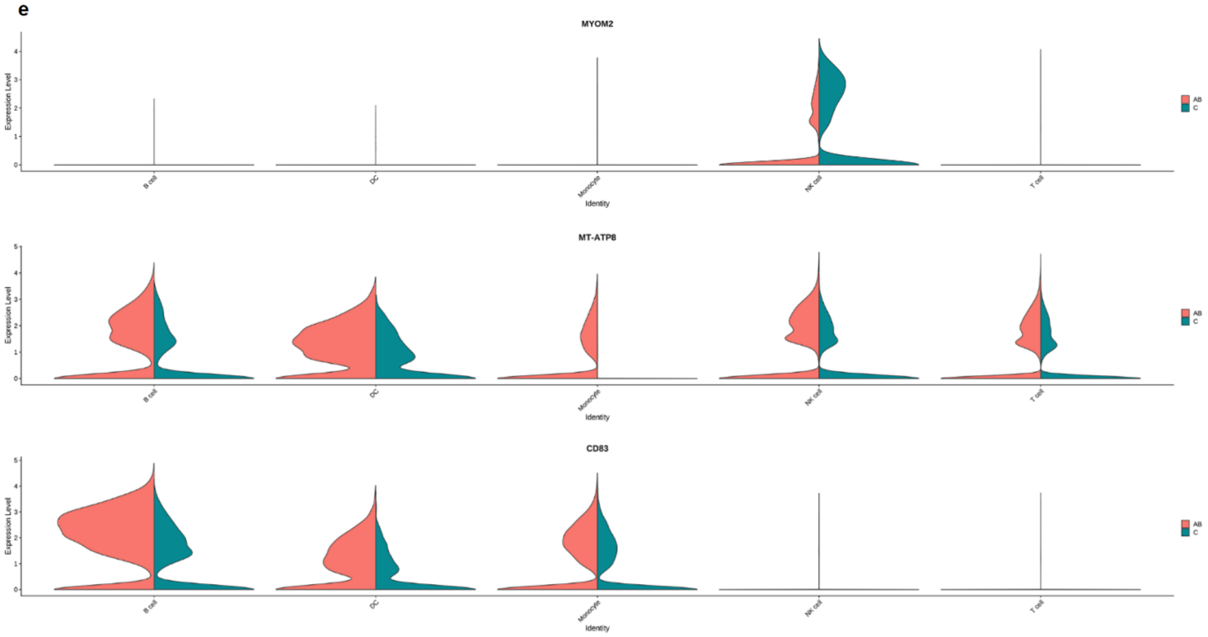
MYOM2
Expression Level
B cell
DC
Monocyte
NK cell
T cell
Identity
AB
C
MT-ATP8
CD83

Fig. S3

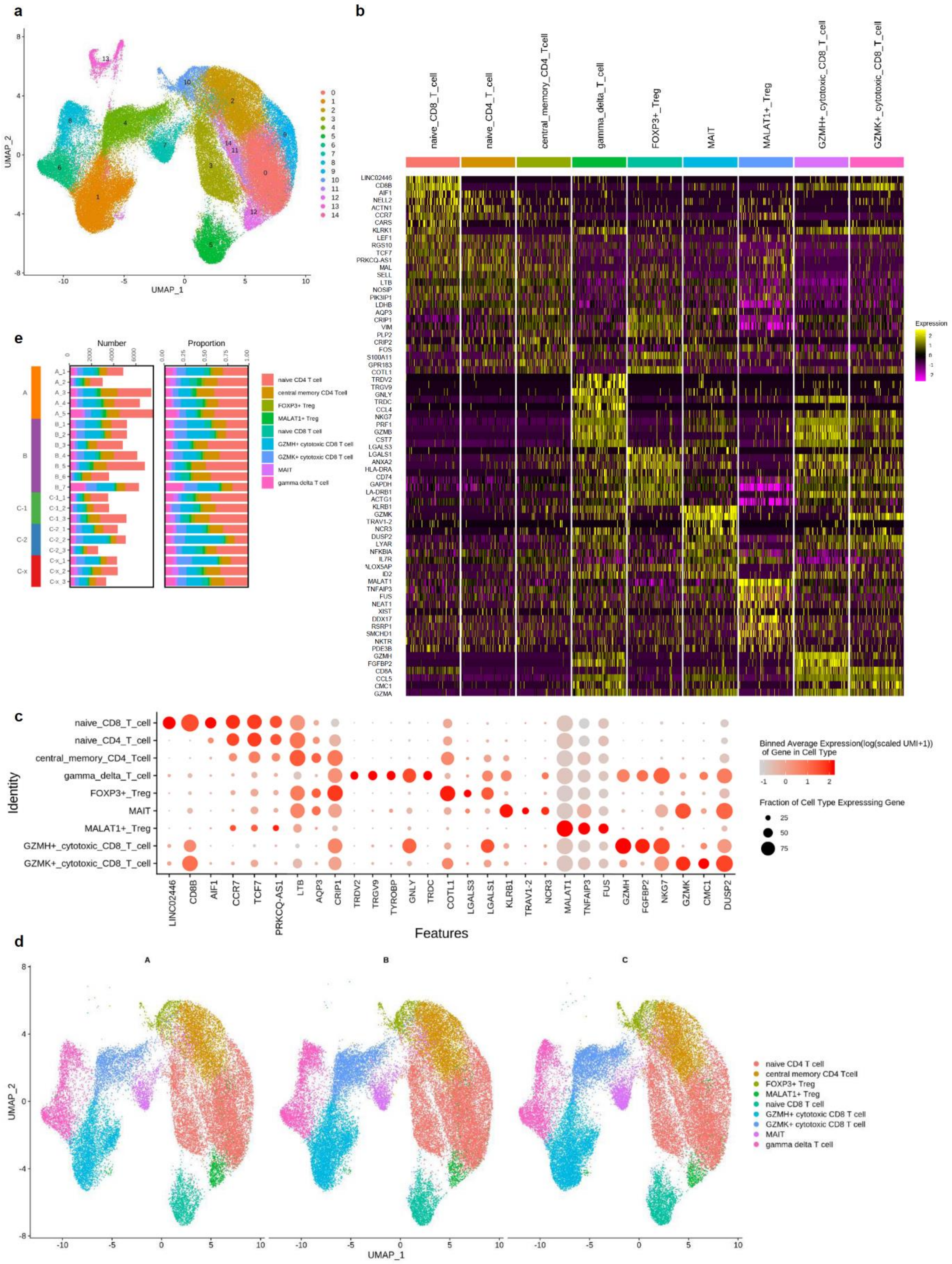
a
b
c
d
e
UMAP_1
UMAP_2
naive_CD8_T_cell
naive_CD4_T_cell
central_memory_CD4_Tcell
gamma_delta_T_cell
FOXP3+_Treg
MAIT
MALAT1+_Treg
GZMH+_cytotoxic_CD8_T_cell
GZMK+_cytotoxic_CD8_T_cell
Expression
Identity
Features
Binned Average Expression(log(scaled UMI+1)) of Gene in Cell Type
Fraction of Cell Type Expresssing Gene
Number
Proportion
naive CD4 T cell
central memory CD4 Tcell
FOXP3+ Treg
MALAT1+ Treg
naive CD8 T cell
GZMH+ cytotoxic CD8 T cell
GZMK+ cytotoxic CD8 T cell
MAIT
gamma delta T cell
A
B
C

Fig. S4

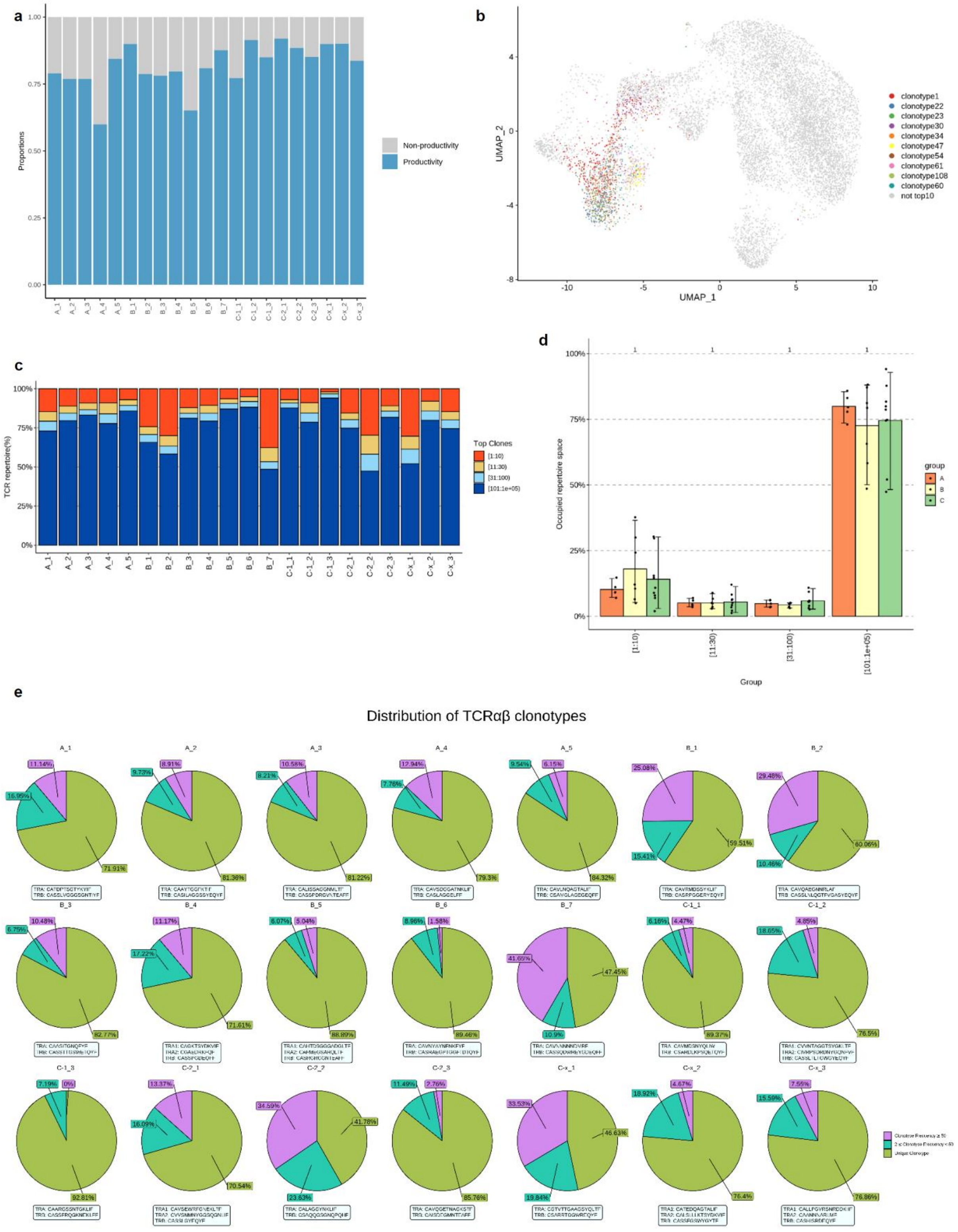
a
Proportions
Non-productivity
Productivity
b
UMAP_1
UMAP_2
clonotype1
clonotype22
clonotype23
clonotype30
clonotype34
clonotype47
clonotype54
clonotype61
clonotype108
clonotype60
not top10
c
TCR repertoire(%)
Top Clones
[1:10)
[11:30)
[31:100)
[101:1e+05)
d
Occupied repertoire space
Group
group
A
B
C
e
Distribution of TCRαβ clonotypes

Fig. S5

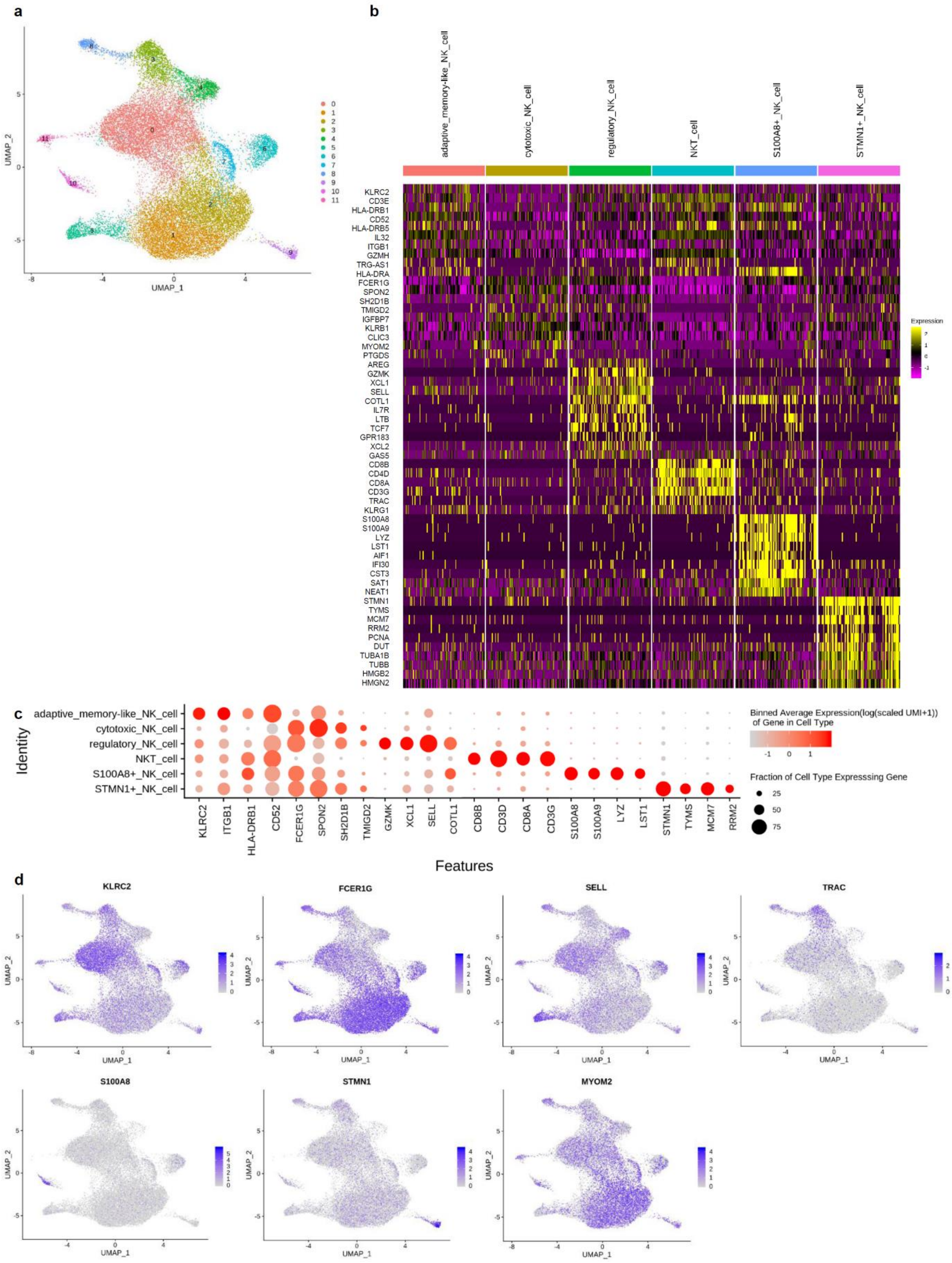
a
b
c
d
UMAP_1
UMAP_2
adaptive_memory-like_NK_cell
cytotoxic_NK_cell
regulatory_NK_cell
NKT_cell
S100A8+_NK_cell
STMN1+_NK_cell
Expression
Identity
Features
Binned Average Expression(log(scaled UMI+1)) of Gene in Cell Type
Fraction of Cell Type Expresssing Gene
KLRC2
FCER1G
SELL
TRAC
S100A8
STMN1
MYOM2

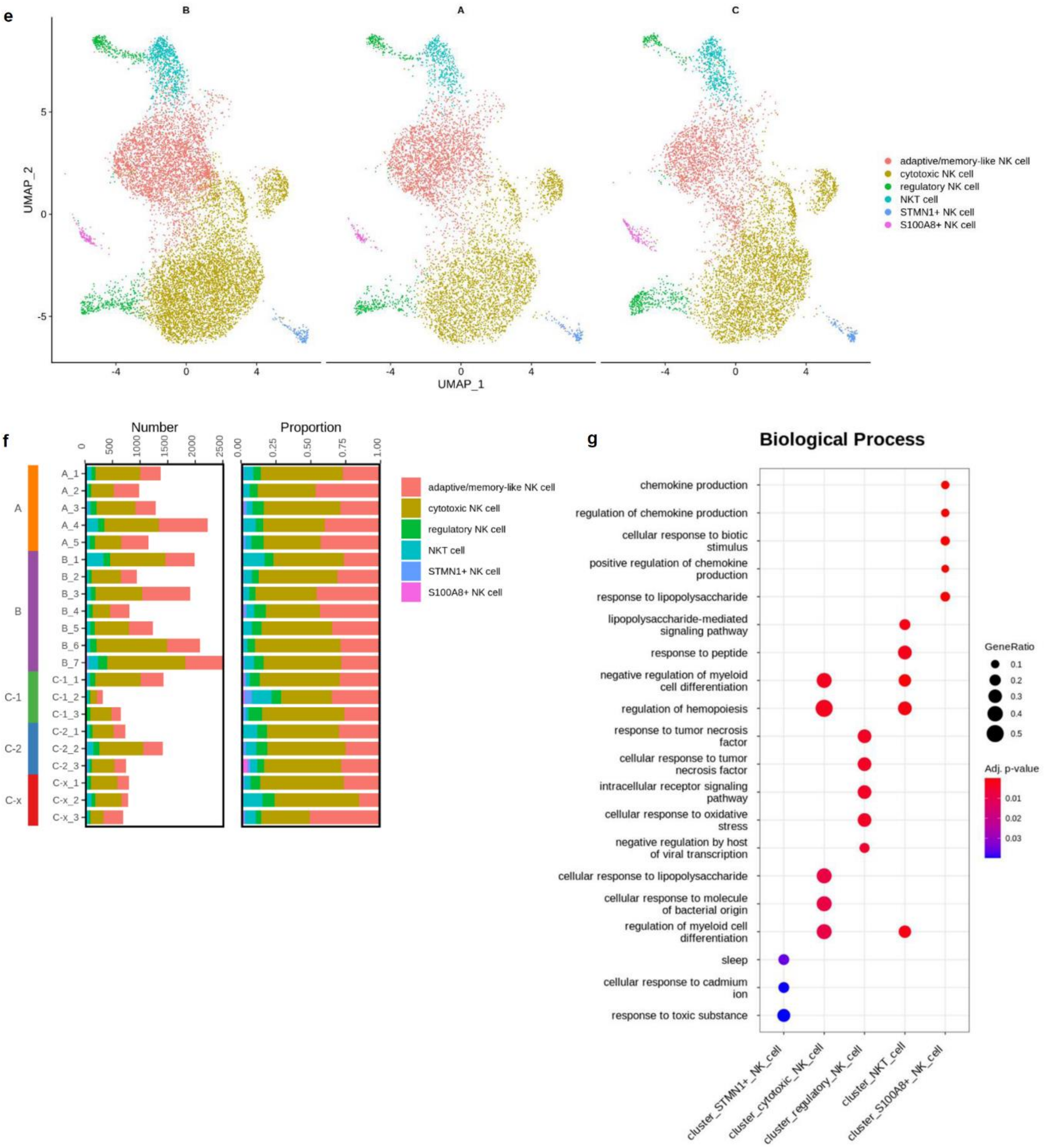
e
B
A
C
UMAP_2
UMAP_1
adaptive/memory-like NK cell
cytotoxic NK cell
regulatory NK cell
NKT cell
STMN1+ NK cell
S100A8+ NK cell
f
Number
Proportion
A
B
C-1
C-2
C-x
A_1
A_2
A_3
A_4
A_5
B_1
B_2
B_3
B_4
B_5
B_6
B_7
C-1_1
C-1_2
C-1_3
C-2_1
C-2_2
C-2_3
C-x_1
C-x_2
C-x_3
adaptive/memory-like NK cell
cytotoxic NK cell
regulatory NK cell
NKT cell
STMN1+ NK cell
S100A8+ NK cell
g
Biological Process
chemokine production
regulation of chemokine production
cellular response to biotic stimulus
positive regulation of chemokine production
response to lipopolysaccharide
lipopolysaccharide-mediated signaling pathway
response to peptide
negative regulation of myeloid cell differentiation
regulation of hemopoiesis
response to tumor necrosis factor
cellular response to tumor necrosis factor
intracellular receptor signaling pathway
cellular response to oxidative stress
negative regulation by host of viral transcription
cellular response to lipopolysaccharide
cellular response to molecule of bacterial origin
regulation of myeloid cell differentiation
sleep
cellular response to cadmium ion
response to toxic substance
GeneRatio
Adj. p-value
cluster_STMN1+_NK_cell
cluster_cytotoxic_NK_cell
cluster_regulatory_NK_cell
cluster_NKT_cell
cluster_S100A8+_NK_cell

h

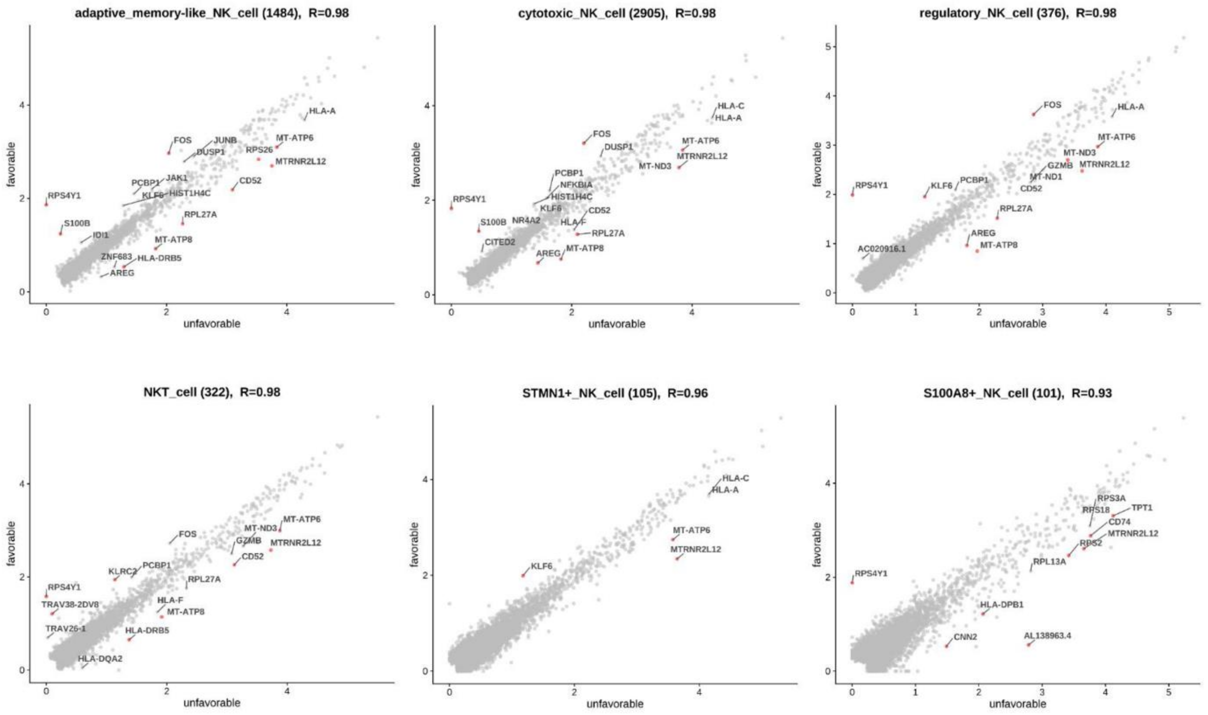

adaptive_memory-like_NK_cell (1484), R=0.98
cytotoxic_NK_cell (2905), R=0.98
regulatory_NK_cell (376), R=0.98
NKT_cell (322), R=0.98
STMN1+_NK_cell (105), R=0.96
S100A8+_NK_cell (101), R=0.93
favorable
unfavorable
HLA-A
HLA-C
FOS
JUNB
DUSP1
MT-ATP6
RPS26
MTRNR2L12
JAK1
PCBP1
KLF6
HIST1H4C
CD52
RPS4Y1
RPL27A
S100B
IDI1
MT-ATP8
ZNF683
HLA-DRB5
AREG
MT-ND3
NFKBIA
NR4A2
HLA-F
CITED2
GZMB
MT-ND1
AC020916.1
KLRC2
TRAV38-2DV8
TRAV26-1
HLA-DQA2
RPS3A
RPS18
TPT1
CD74
RPS2
RPL13A
HLA-DPB1
CNN2
AL138963.4

i

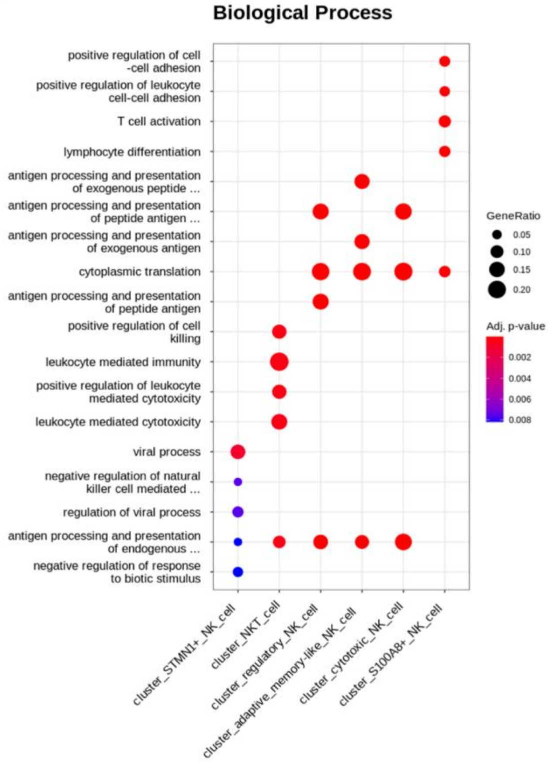

Biological Process
positive regulation of cell -cell adhesion
positive regulation of leukocyte cell-cell adhesion
T cell activation
lymphocyte differentiation
antigen processing and presentation of exogenous peptide ...
antigen processing and presentation of peptide antigen ...
antigen processing and presentation of exogenous antigen
cytoplasmic translation
antigen processing and presentation of peptide antigen
positive regulation of cell killing
leukocyte mediated immunity
positive regulation of leukocyte mediated cytotoxicity
leukocyte mediated cytotoxicity
viral process
negative regulation of natural killer cell mediated ...
regulation of viral process
antigen processing and presentation of endogenous ...
negative regulation of response to biotic stimulus
GeneRatio
0.05
0.10
0.15
0.20
Adj. p-value
0.002
0.004
0.006
0.008
cluster_STMN1+_NK_cell
cluster_NKT_cell
cluster_regulatory_NK_cell
cluster_adaptive_memory-like_NK_cell
cluster_cytotoxic_NK_cell
cluster_S100A8+_NK_cell

Fig. S6

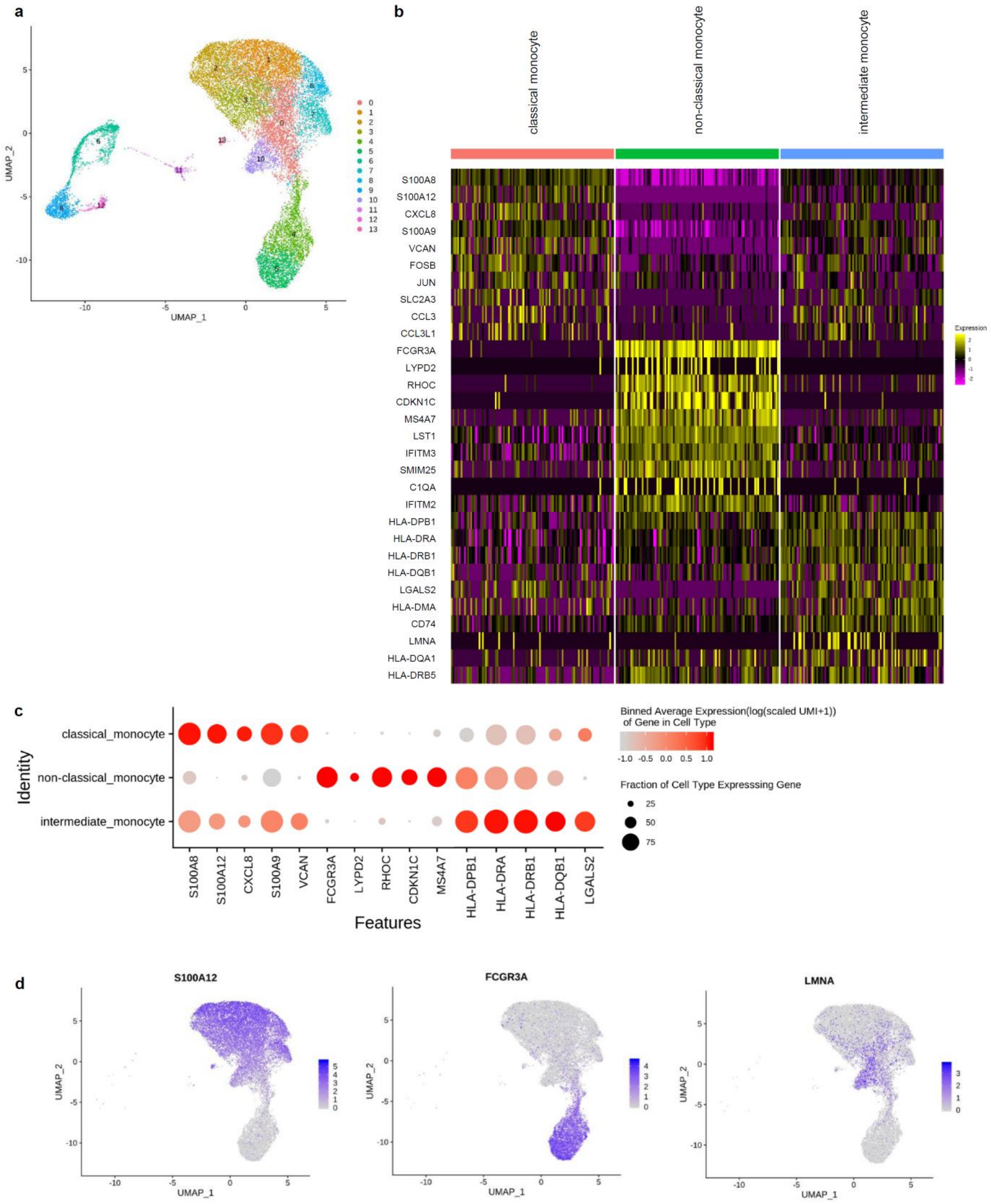
a
b
c
d
UMAP_1
UMAP_2
classical monocyte
non-classical monocyte
intermediate monocyte
S100A8
S100A12
CXCL8
S100A9
VCAN
FOSB
JUN
SLC2A3
CCL3
CCL3L1
FCGR3A
LYPD2
RHOC
CDKN1C
MS4A7
LST1
IFITM3
SMIM25
C1QA
IFITM2
HLA-DPB1
HLA-DRA
HLA-DRB1
HLA-DQB1
LGALS2
HLA-DMA
CD74
LMNA
HLA-DQA1
HLA-DRB5
Expression
Identity
classical_monocyte
non-classical_monocyte
intermediate_monocyte
Features
Binned Average Expression(log(scaled UMI+1))
of Gene in Cell Type
Fraction of Cell Type Expresssing Gene
S100A12
FCGR3A
LMNA

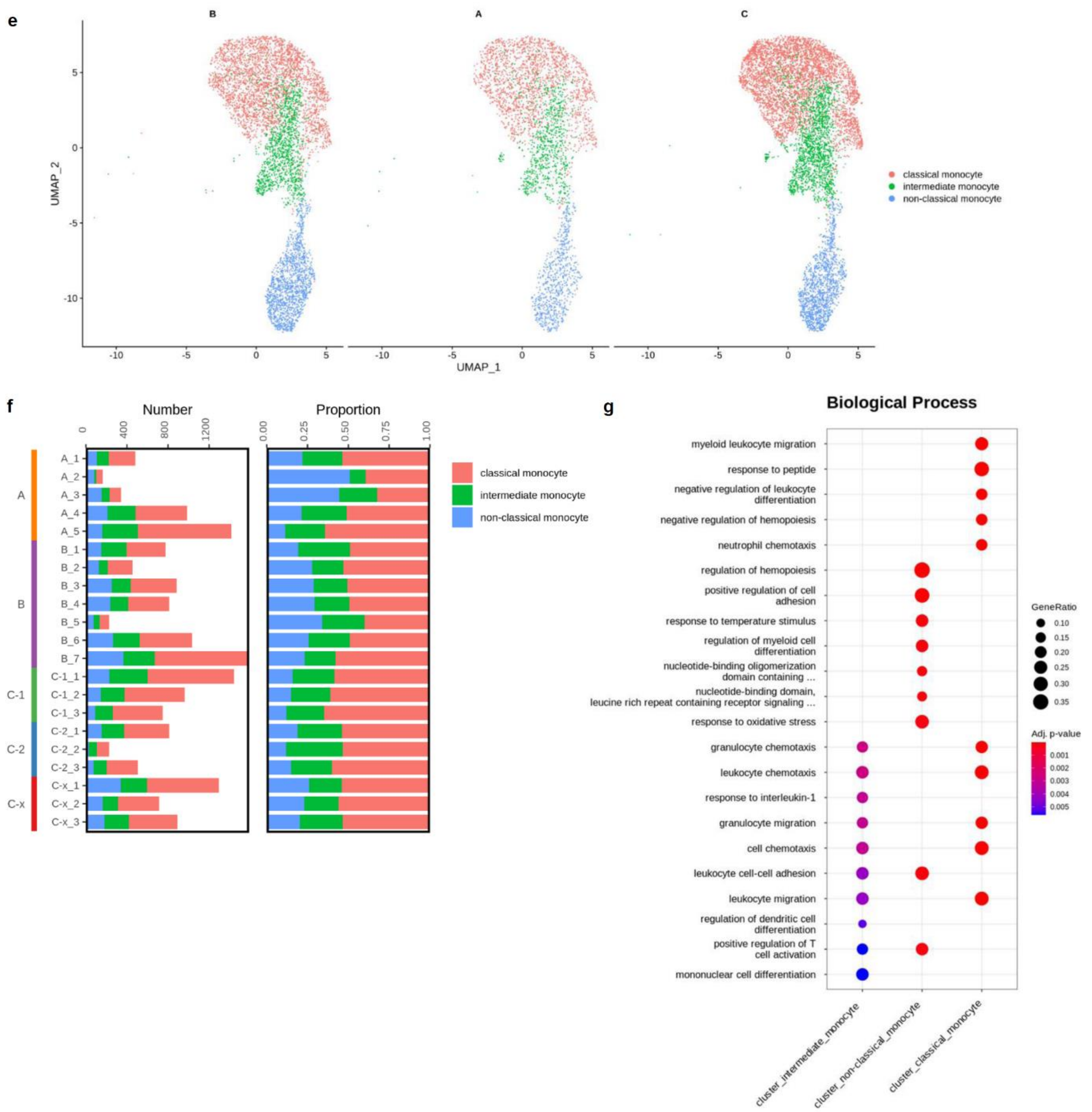
e
B
A
C
UMAP_2
UMAP_1
classical monocyte
intermediate monocyte
non-classical monocyte
f
Number
Proportion
A
B
C-1
C-2
C-x
classical monocyte
intermediate monocyte
non-classical monocyte
g
Biological Process
myeloid leukocyte migration
response to peptide
negative regulation of leukocyte differentiation
negative regulation of hemopoiesis
neutrophil chemotaxis
regulation of hemopoiesis
positive regulation of cell adhesion
response to temperature stimulus
regulation of myeloid cell differentiation
nucleotide-binding oligomerization domain containing ...
nucleotide-binding domain, leucine rich repeat containing receptor signaling ...
response to oxidative stress
granulocyte chemotaxis
leukocyte chemotaxis
response to interleukin-1
granulocyte migration
cell chemotaxis
leukocyte cell-cell adhesion
leukocyte migration
regulation of dendritic cell differentiation
positive regulation of T cell activation
mononuclear cell differentiation
GeneRatio
Adj. p-value
cluster_intermediate_monocyte
cluster_non-classical_monocyte
cluster_classical_monocyte

Fig. S7

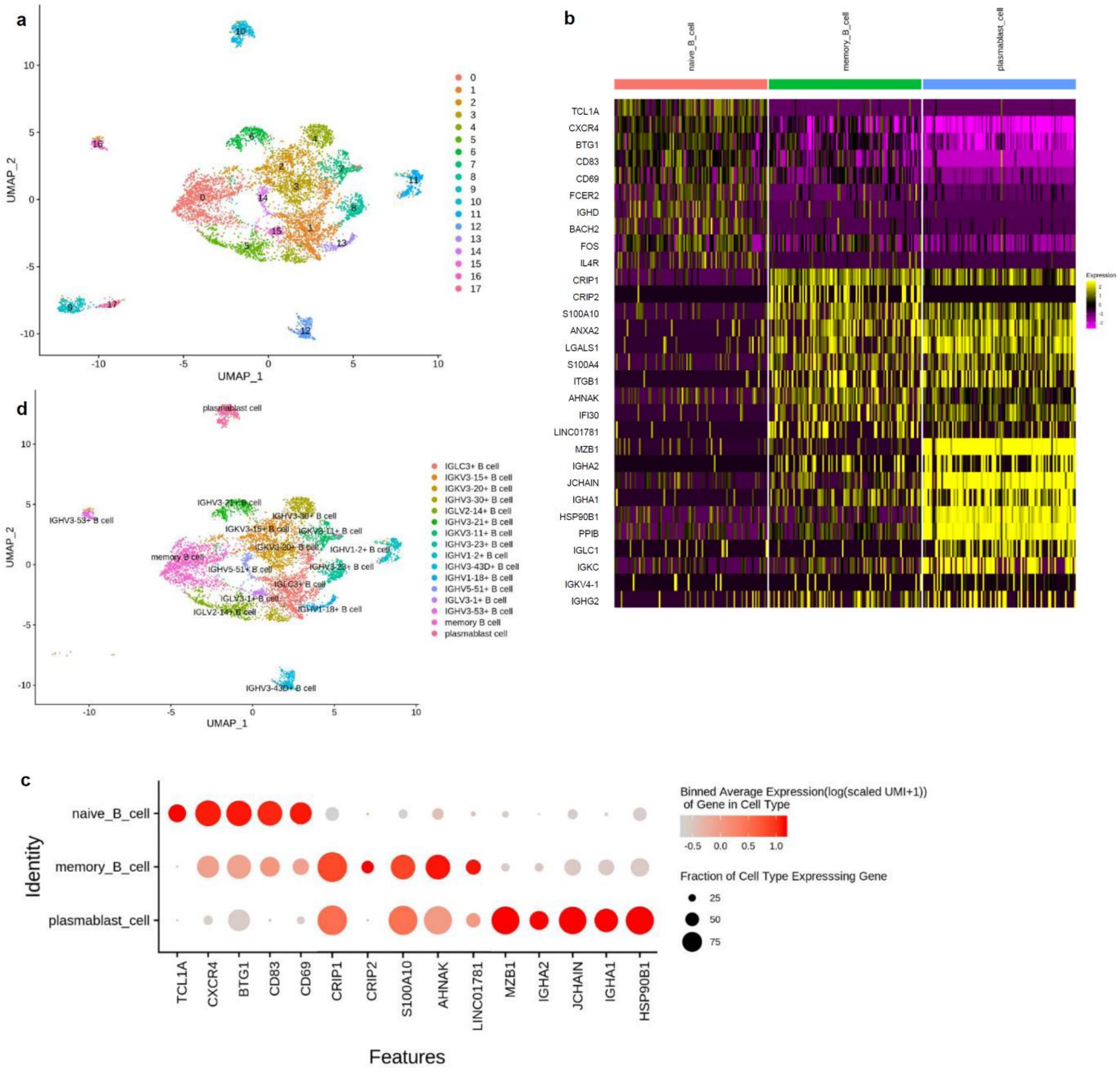
a
b
c
d
UMAP_1
UMAP_2
naive_B_cell
memory_B_cell
plasmablast_cell
Expression
TCL1A
CXCR4
BTG1
CD83
CD69
FCER2
IGHD
BACH2
FOS
IL4R
CRIP1
CRIP2
S100A10
ANXA2
LGALS1
S100A4
ITGB1
AHNAK
IFI30
LINC01781
MZB1
IGHA2
JCHAIN
IGHA1
HSP90B1
PPIB
IGLC1
IGKC
IGKV4-1
IGHG2
IGLC3+ B cell
IGKV3-15+ B cell
IGKV3-20+ B cell
IGHV3-30+ B cell
IGLV2-14+ B cell
IGHV3-21+ B cell
IGKV3-11+ B cell
IGHV3-23+ B cell
IGHV1-2+ B cell
IGHV3-43D+ B cell
IGHV1-18+ B cell
IGHV5-51+ B cell
IGLV3-1+ B cell
IGHV3-53+ B cell
memory B cell
plasmablast cell
Identity
Features
Binned Average Expression(log(scaled UMI+1)) of Gene in Cell Type
Fraction of Cell Type Expresssing Gene

e

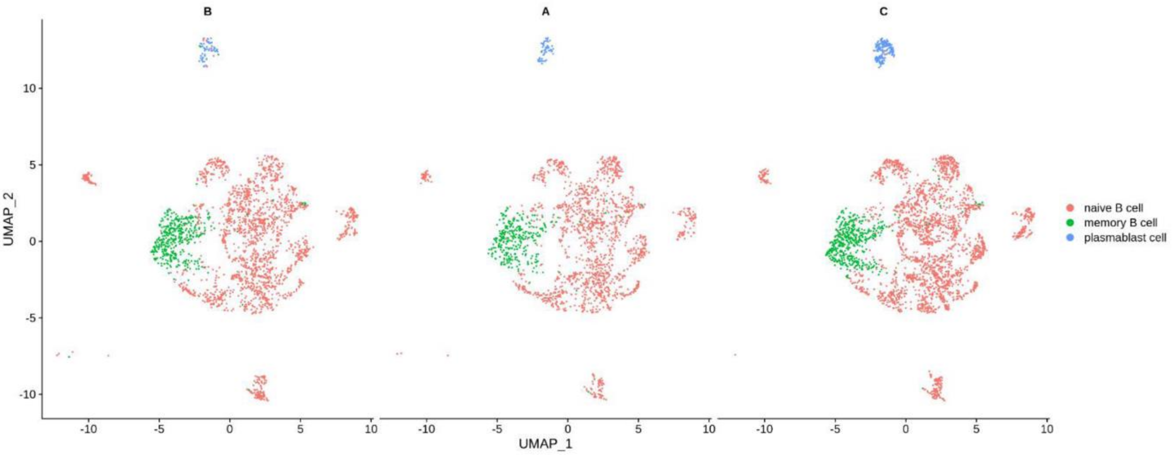
B
A
C
UMAP_2
UMAP_1
naive B cell
memory B cell
plasmablast cell

f

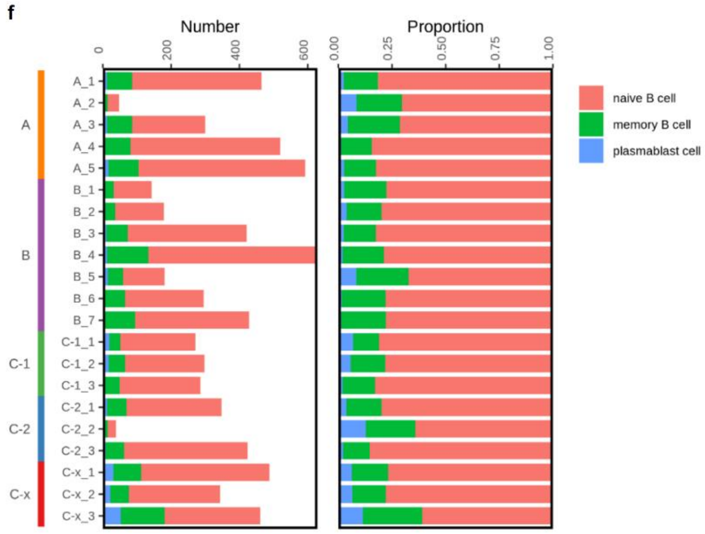
Number
Proportion
A
B
C-1
C-2
C-x
naive B cell
memory B cell
plasmablast cell

g

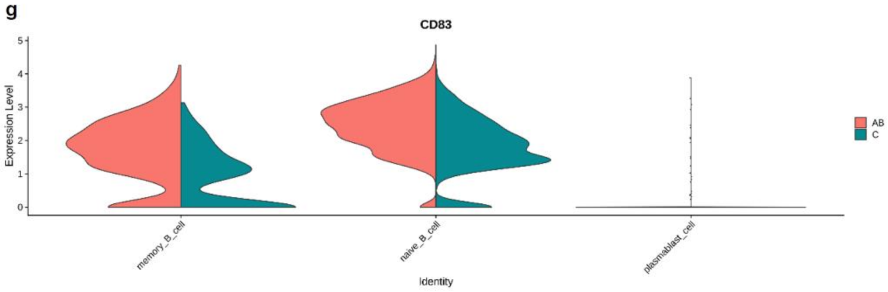
CD83
Expression Level
memory_B_cell
naive_B_cell
plasmablast_cell
Identity
AB
C

Fig. S8

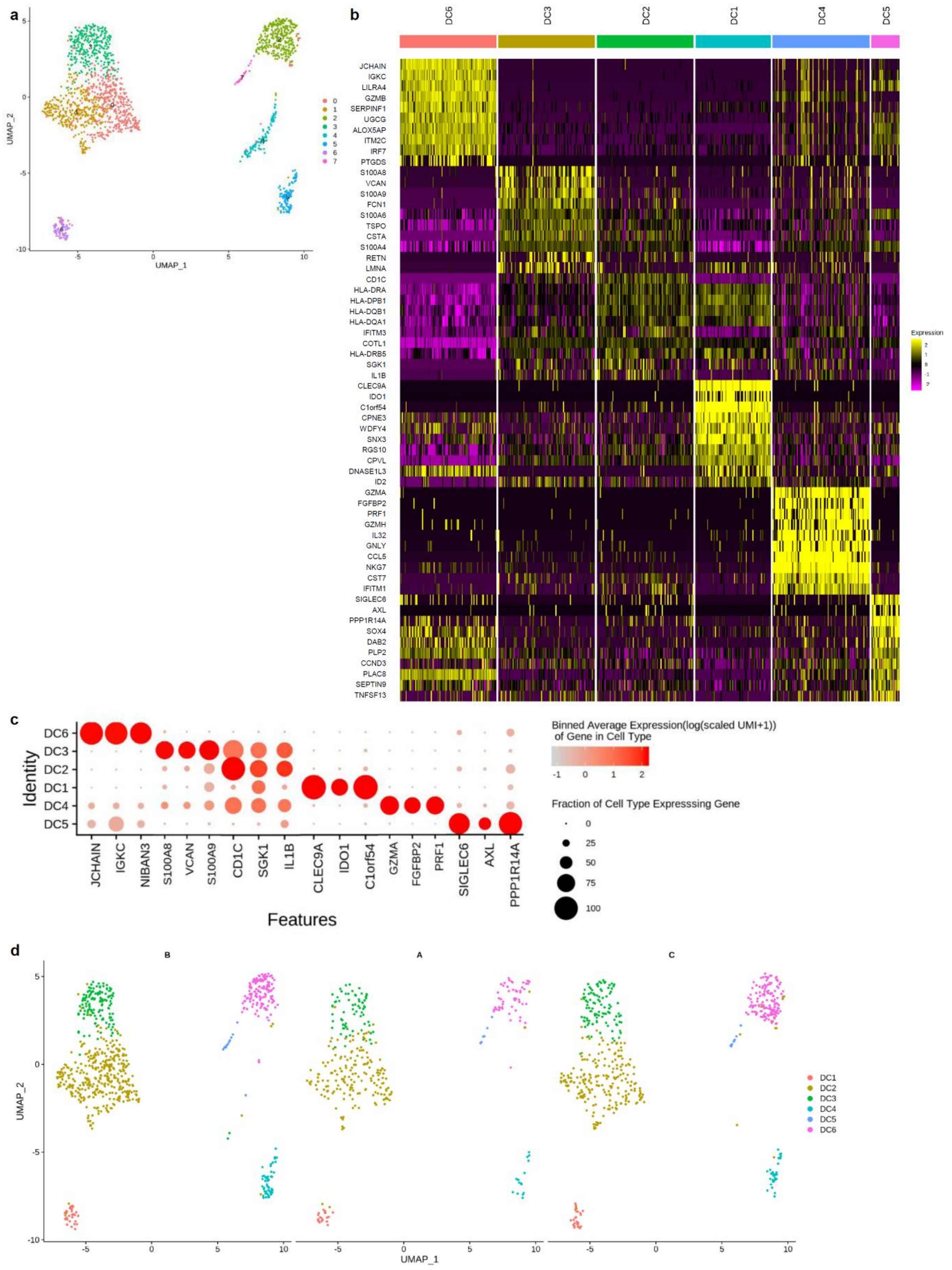
a
UMAP_1
UMAP_2
b
DC6
DC3
DC2
DC1
DC4
DC5
Expression
JCHAIN
IGKC
LILRA4
GZMB
SERPINF1
UGCG
ALOX5AP
ITM2C
IRF7
PTGDS
S100A8
VCAN
S100A9
FCN1
S100A6
TSPO
CSTA
S100A4
RETN
LMNA
CD1C
HLA-DRA
HLA-DPB1
HLA-DQB1
HLA-DQA1
IFITM3
COTL1
HLA-DRB5
SGK1
IL1B
CLEC9A
IDO1
C1orf54
CPNE3
WDFY4
SNX3
RGS10
CPVL
DNASE1L3
ID2
GZMA
FGFBP2
PRF1
GZMH
IL32
GNLY
CCL5
NKG7
CST7
IFITM1
SIGLEC6
AXL
PPP1R14A
SOX4
DAB2
PLP2
CCND3
PLAC8
SEPTIN9
TNFSF13
c
Identity
Features
Binned Average Expression(log(scaled UMI+1)) of Gene in Cell Type
Fraction of Cell Type Expresssing Gene
JCHAIN
IGKC
NIBAN3
S100A8
VCAN
S100A9
CD1C
SGK1
IL1B
CLEC9A
IDO1
C1orf54
GZMA
FGFBP2
PRF1
SIGLEC6
AXL
PPP1R14A
d
B
A
C
UMAP_1
UMAP_2
DC1
DC2
DC3
DC4
DC5
DC6

e

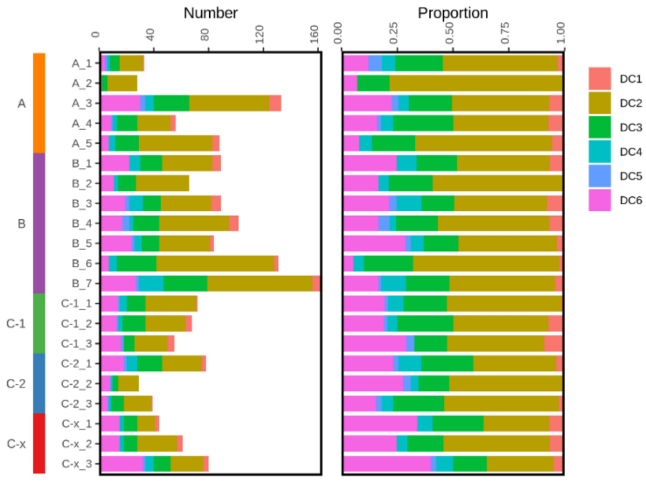
Number
Proportion
0
40
80
120
160
0.00
0.25
0.50
0.75
1.00
A_1
A_2
A_3
A_4
A_5
B_1
B_2
B_3
B_4
B_5
B_6
B_7
C-1_1
C-1_2
C-1_3
C-2_1
C-2_2
C-2_3
C-x_1
C-x_2
C-x_3
A
B
C-1
C-2
C-x
DC1
DC2
DC3
DC4
DC5
DC6
