# Supplementary material for: Single-cell transcriptomics by clinical course of Mycobacterium avium complex pulmonary disease
Source: Sci Rep. 2024 Jul 8;14:15663. doi: 10.1038/s41598-024-66523-x (PMC11231222; doi:10.1038/s41598-024-66523-x)
Supplement: Supplementary file 3 — Supplementary Information 3. [file 41598_2024_66523_MOESM3_ESM.pdf]

```
suppressPackageStartupMessages(require(Seurat))

suppressPackageStartupMessages(require(dplyr))

options(bitmapType='cairo', future.rng.onMisuse="ignore")


k <- 30


test.data <- Read10X(data.dir=input.path)

test.obj <- CreateSeuratObject(counts = test.data, project = 'mouse', min.cells = 3, min.features = 200)

test.obj[["percent.mt"]] <- PercentageFeatureSet(test.obj, pattern="^MT-|^mt-|^Mt-")

test.obj <- subset(test.obj, subset=nFeature_RNA > 200 & nFeature_RNA < 9000 & percent.mt < 10)


# Seurat(anchor and CCA)

        # anchoring procedure consists of four broad steps, as explained in detail below:

        # (1) data preprocessing and feature selection,

        # (2) dimension reduction and identification of "anchor" correspondences between datasets,

        # (3) filtering, scoring, and weighting of anchor correspondences,

        # (4) data matrix correction, or data transfer across experiments.


test.obj.list <- SplitObject(test.obj, split.by="batch")

  for(i in 1:length(test.obj.list)) {

    test.obj.list[[i]] <- NormalizeData(test.obj.list[[i]], verbose=FALSE)

    test.obj.list[[i]] <- FindVariableFeatures(test.obj.list[[i]], selection.method="vst",  nfeatures=2000, verbose=FALSE)

  }
```

```
features <- SelectIntegrationFeatures(object.list = test.obj.list)

test.obj.anchors <- FindIntegrationAnchors(test.obj.list, dims=1:k)

test.integrated <- IntegrateData(anchorset=test.obj.anchors,dims=1:k)


DefaultAssay(test.integrated) <- 'integrated'

test.integrated <- ScaleData(test.integrated, verbose=FALSE,vars.to.regress="percent.mt")


test.integrated <- RunPCA(test.integrated, npcs=k)

test.integrated <- RunUMAP(test.integrated, dims = 1:k)


test.integrated <- FindNeighbors(test.integrated, dims = 1:k)

test.integrated <- FindClusters(test.integrated, resolution = 0.5)



### Finding differentially expressed features(cluster biomarkers)

test.obj <- test.integrated


### FindAllMarkers

test.obj.markers <- FindAllMarkers(object=test.obj, only.pos=TRUE, min.pct=0.25, logfc.threshold=0.25)



test.obj.markers %>% group_by(cluster) %>% top_n(20, avg_log2FC) -> top20_list
```

```
genelist <- top20_list$gene

FeaturePlot(object=test.obj, features=genelist, cols= c("lightgrey","blue") , reduction=reduction_method,order=TRUE, raster=FALSE)

## top10 genes

test.obj.markers %>% group_by(cluster) %>% top_n(10, avg_log2FC) -> top10

DoHeatmap(object=temp.obj, features=top10$gene, angle=90)+ theme(axis.text.y=element_text(size=5))+ guides(colour=guide_colourbar())

### Finding differentially expressed genes between two groups for each cluster

group.name <- c("C_vs_AB")

split.by <- "group"

group.by="seurat_clusters"

case.group<-unlist(strsplit(group.name, "_vs_"))[1]

control.group <-unlist(strsplit(group.name, "_vs_"))[2]

### FindMarkers

temp.result <-FindMarkers(test.obj, ident.1=group1, ident.2=group2, logfc.threshold=0, assay=DefaultAssay(test.obj))
```

```
match.pos <- match(rownames(temp.result),anno$gene.symbol, nomatch=0)

Bycluster <-
levels(as.factor(test.obj@meta.data[,which(colnames(test.obj@meta.data)==clust.colname)]))

sub.markers<-cbind(cluster=Bycluster[i],gene=rownames(temp.result),temp.result,anno[match.pos,])
sub.markers$fc <- fc(sub.markers$avg_log2FC)
col.match <-match(c("gene_id", "gene_name","Entrez_Gene_ID", "gene_biotype",
"p_val","avg_log2FC", "pct.1", "pct.2", "p_val_adj", "cluster", "gene","fc") , colnames(sub.markers),
nomatch=0)

temp.data <- sub.markers[,col.match]
temp.sig.pos<-abs(temp.data$fc) >=1.5

write.table(temp.data, file=paste0(Out.path, "/DEGbyGroup/",group.name,"/cluster", Bycluster[i],
".txt"), sep="₩t", col.names=T, row.names=F, quote=F)



## Dot plot
temp.cells<- WhichCells(object = test.obj, idents = c(case.group, control.group))
combi.test.obj <-subset(test.obj, cells=temp.cells)
combi.test.obj@meta.data[,split.by] <- as.factor(as.vector(combi.test.obj@meta.data[,split.by]))
Idents(combi.test.obj) <- split.by

rev.factor <- rev(levels(Idents(combi.test.obj)))
temp.match.pos <-match(levels(Idents(combi.test.obj)), rev.factor)
```

```
Idents(combi.test.obj)<-factor(Idents(combi.test.obj),label= temp.match.pos)

Idents(combi.test.obj)<-factor(as.numeric(as.vector(Idents(combi.test.obj))))

Idents(combi.test.obj)<-factor(Idents(combi.test.obj), label=rev.factor)


png(filename=paste0(plot.path, '/DotPlot_',title.name,'_by_',split.by,'.png'), width=width.size, height=height.size, res=300)

	p1 <- DotPlot(combi.test.obj, features = genes.to.plot, dot.scale = 8, cols = c("#f36e62", "#6c5aa6"),split.by = split.by)+ RotatedAxis()

	p1 <- p1 + guides(size = guide_legend(title="Fraction of Cell Type Expresssing Gene"), title.position="top", direction="horizontal")

	p1 <- p1 + scale_x_discrete(limits = as.vector(genes.to.plot))

	p1 <- p1 + theme(axis.text.x = element_text(angle = 90), axis.text=element_text(size=rel(0.7)), legend.title=element_text(size=rel(0.6)), legend.text=element_text(size=rel(0.5)), legend.justification="center")

	print(patchwork::wrap_plots(p1))

dev.off()




### VlnPlot

png(filename=paste0(plot.path, '/VlnPlot_target_gene_', title.name, '.png'), width=width.size, height=1500, res=300)

	plots<-VlnPlot(combi.test.obj, features=gene.to.plot, split.by=split.by, group.by=group.by, pt.size=0, combine=FALSE,split.plot = TRUE )

	print(patchwork::wrap_plots(plots, ncol=1))


dev.off()
```

```r
## ScatterPlot

library(ggplot2)
library(cowplot)
theme_set(theme_cowplot())

posi.pos <-sub.markers$p_val_adj < 0.05 & sub.markers$fc>=2
neg.pos <-sub.markers$p_val_adj < 0.05 & sub.markers$fc<=-2

pos <- posi.pos==TRUE | neg.pos==TRUE

sig.posi.markers <- sub.markers.mat[posi.pos,]
sig.neg.markers <- sub.markers.mat[neg.pos,]

dplyr::arrange(sig.posi.markers, cluster, desc(fc),p_val_adj) %>% group_by(cluster) %>% top_n(10, fc) -> top10
dplyr::arrange(sig.neg.markers, cluster,fc, p_val_adj) %>% group_by(cluster) %>% top_n(-10, fc) -> bottom10
sig_top20 <- rbind(top10, bottom10)

genes.to.label <-as.vector(sig_top20$gene[sig_top20$cluster==clusterName[j]])
```

```
case.group<-unlist(strsplit(group.name, "_vs_"))[1]
control.group <-unlist(strsplit(group.name, "_vs_"))[2]

sub.clst.cells <- subset(test.obj, idents=cluster.name)

Idents(sub.clst.cells) <- "group"
avg.sub.clst.cells <- log1p(eval(parse(text= paste0("AverageExpression(sub.clst.cells,assays='",DefaultAssay(sub.clst.cells),"', verbose = FALSE)$",DefaultAssay(sub.clst.cells)))))
avg.sub.clst.cells<-data.frame(cbind(avg.sub.clst.cells, gene= rownames(avg.sub.clst.cells)))
colnames(avg.sub.clst.cells) <- c("CTRL", "TEST", "gene")

avg.sub.clst.cells[,1:2] <- apply(avg.sub.clst.cells[,1:2],2,as.numeric)
sig.pos <- abs(data.mat$fc)>2 & data.mat$p_val_adj < 0.05
	col <-c()
	col[sig.pos] <- "sig"
	col[!sig.pos] <- "not.sig"
	col<-as.matrix(col)
	rownames(col) <- rownames(data.mat)

	avg.sub.clst.cells <- cbind(avg.sub.clst.cells[match(data.mat$gene, rownames(avg.sub.clst.cells),nomatch=0),-match("gene",colnames(avg.sub.clst.cells), nomatch=0)],gene=data.mat[,"gene"], col=as.matrix(col))

	cor.result<-cor.test(avg.sub.clst.cells$CTRL, avg.sub.clst.cells$TEST)
	coeff<-as.numeric(cor.result$estimate)
	ncells <- dim(sub.clst.cells)[2]
```

```
p1 <- ggplot(avg.sub.clst.cells, aes(CTRL,TEST, color=col)) +
        geom_point(alpha=1/2) + scale_color_manual(values=c("gray", "red")) +
        ggtitle(paste0(title.cluster.name, " (",ncells,")",", R = ",roundup(coeff,2))) +
        labs(x=control.group, y=case.group)
        if(length(genes.to.label) >0){
                p1 <- LabelPoints(plot = p1, points = genes.to.label, repel = TRUE,
                        fontface = 'bold', color="grey27", xnudge=0.3, ynudge=0.2) + theme(plot.title = element_text(hjust = 0.5)) + guides(color=FALSE)
        }
ggsave(file=paste0(plot.path, '/ScatterPlot_cluster', cluster.name, '_by_group.png'), plot=p1, dpi=600, width=18, height=15, unit='cm', bg="white")
```
